# *RB1*-deficient prostate tumor growth and metastasis are vulnerable to ferroptosis induction via the E2F/ACSL4 axis

**DOI:** 10.1172/JCI166647

**Published:** 2023-05-15

**Authors:** Mu-En Wang, Jiaqi Chen, Yi Lu, Alyssa R. Bawcom, Jinjin Wu, Jianhong Ou, John M. Asara, Andrew J. Armstrong, Qianben Wang, Lei Li, Yuzhuo Wang, Jiaoti Huang, Ming Chen

**Affiliations:** 1 Department of Pathology, Duke University School of Medicine, Durham, North Carolina, USA.; 2 Duke Cancer Institute, Duke University, Durham, North Carolina, USA.; 3 Department of Urology, The First Affiliated Hospital of Xi’an Jiaotong University, Xi’an, China.; 4 Regeneration Center, Duke University, Durham, North Carolina, USA.; 5 Division of Signal Transduction, Beth Israel Deaconess Medical Center and Department of Medicine, Harvard Medical School, Boston, Massachusetts, USA.; 6 Department of Pharmacology and Cancer Biology, Department of Medicine, Division of Medical Oncology and Urology, Duke University School of Medicine, Durham, North Carolina, USA.; 7 Center for Prostate and Urologic Cancers, Duke Cancer Institute, Duke University, Durham, North Carolina, USA.; 8 Vancouver Prostate Centre, Vancouver General Hospital and Department of Urologic Sciences, The University of British Columbia, Vancouver, British Columbia, Canada.; 9 Department of Experimental Therapeutics, BC Cancer Research Centre, Vancouver, British Columbia, Canada.

**Keywords:** Oncology, Prostate cancer

## Abstract

Inactivation of the *RB1* tumor suppressor gene is common in several types of therapy-resistant cancers, including metastatic castration-resistant prostate cancer, and predicts poor clinical outcomes. Effective therapeutic strategies against *RB1*-deficient cancers remain elusive. Here, we showed that *RB1* loss/E2F activation sensitized cancer cells to ferroptosis, a form of regulated cell death driven by iron-dependent lipid peroxidation, by upregulating expression of *ACSL4* and enriching ACSL4-dependent arachidonic acid–containing phospholipids, which are key components of ferroptosis execution. *ACSL4* appeared to be a direct E2F target gene and was critical to *RB1* loss–induced sensitization to ferroptosis. Importantly, using cell line–derived xenografts and genetically engineered tumor models, we demonstrated that induction of ferroptosis in vivo by JKE-1674, a highly selective and stable GPX4 inhibitor, blocked *RB1*-deficient prostate tumor growth and metastasis and led to improved survival of the mice. Thus, our findings uncover an RB/E2F/ACSL4 molecular axis that governs ferroptosis and also suggest a promising approach for the treatment of *RB1*-deficient malignancies.

## Graphical abstract

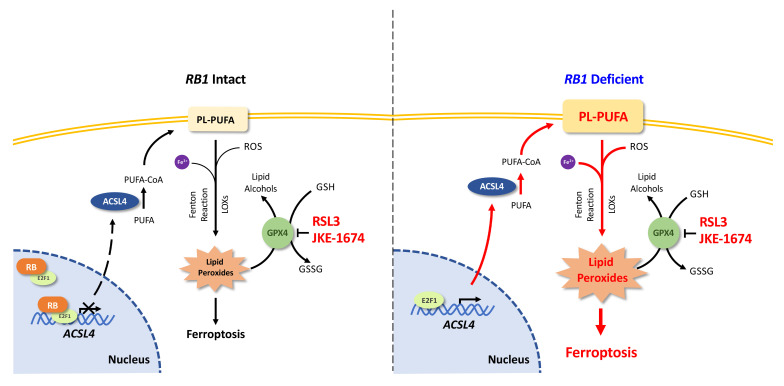

## Introduction

Mutations in the retinoblastoma tumor suppressor gene, *RB1*, are common in a wide spectrum of pediatric and adult cancers, including retinoblastoma, sarcoma, small cell lung and prostate cancers, and carcinomas of the prostate, breast, and bladder (1–4). The retinoblastoma tumor suppressor protein (RB) serves as a key cell cycle regulator that is dephosphorylated and activated in response to myriad antiproliferation stresses, such as antimitogens and therapeutic agents, to restrain members of the E2F family of transcriptional factors (E2F1, E2F2, and E2F3), thereby preventing unscheduled entry into the mitotic cell cycle (2, 3). Conversely, to drive cell proliferation, mitogens and hormones must counteract RB action, specifically through activation of CDK-cyclin complexes, which phosphorylate RB and abolish its binding with E2F and its ability to repress E2F-dependent transcription of various genes required for cell cycle progression (2, 3). The loss of RB function, therefore, allows cells to multiply, even in the absence of external growth signals, and it is likely to render cancer cells refractory to therapeutic agents against upstream mitogenic and hormonal pathways. Indeed, *RB1* inactivation has been linked to cancer initiation and progression (2, 3) and resistance to several targeted therapies, including androgen receptor (AR) antagonists (5–7), ER antagonists (8, 9), CDK4/6 inhibitors (10), and EGFR tyrosine kinase inhibitors (11).

In addition to its well-characterized role in controlling S-phase entry, recent studies have revealed an expanded role for RB in impeding cancer aggressiveness, which has been underscored by results from genetically engineered mouse models, in which *Rb1* loss facilitated lineage plasticity, metastasis, therapy resistance, and lethality of prostate and lung adenocarcinomas initiated by other genetic events (6, 12). In recent findings consistent with results obtained in mice (6, 12), *RB1* inactivation has been identified as the only molecular factor independently predictive of poor survival for men with advanced prostate cancer (13, 14), and it likewise decreases overall survival of patients with cancers of the lung, breast, and bladder (15–17). These findings in mice and humans highlight a pressing need for the identification of therapeutic strategies targeting disease mechanisms driven by *RB1* deficiency to reduce cancer mortality rates.

Ferroptosis is a form of regulated cell death driven by iron-dependent lipid peroxidation, especially of phospholipids containing polyunsaturated fatty acid (PL-PUFA) at the plasma and organellar membranes (18). Because the induction of ferroptosis relies on the peroxidation of PL-PUFA, this acyl-CoA synthetase long-chain family member 4 (ACSL4) enzyme, which catalyzes the activation and membrane incorporation of PUFAs, dictates ferroptosis sensitivity (19–21). Additionally, ferroptosis is suppressed by 4 major pathways that negatively regulate the peroxidation of PL-PUFA: the GPX4/glutathione axis, the FSP1/CoQH_2_ axis, the DHODH/CoQH_2_ axis, and the GCH1/BH_4_ axis (22, 23). Emerging evidence suggests that the tumor-suppressive effects of several conventional cancer therapies, including radiation therapy, chemotherapy, and immunotherapy, are mediated at least in part by ferroptosis (23). Furthermore, ferroptosis is a therapeutically tractable target in cancer (23), and induction of ferroptosis through blockade of lipid peroxide repair networks has shown potent antitumor efficacy against several types of human malignancies in multiple preclinical studies (24–29). Importantly, recent studies have shown that therapy-resistant cancers, in which *RB1* inactivation most commonly occurs (10, 11, 13, 14), are vulnerable to ferroptosis (30, 31), and *RB1* knockdown appears to enhance the sensitivity of liver cancer cells to sorafenib-induced ferroptosis in vitro (32). However, the underlying mechanisms of RB-regulated ferroptosis and whether RB regulates ferroptosis in multiple cancer cell types, including prostate cancer, remain unknown.

In this study, we demonstrate that *RB1* disruptions enhance ferroptosis in cancer cell lines of various histological origins. Mechanistically, *RB1* loss/E2F activation upregulates the levels of ACSL4 and enriches ACSL4-dependent arachidonic acid–containing (AA–containing) phospholipids in *RB1*-deficient cells, leading to increased vulnerability to ferroptosis. Critically, the antitumor and antimetastatic activity exhibited by ferroptosis is significantly higher in prostate cancer lacking functional RB. Thus, our results have potential implications for the treatment of *RB1* loss–driven prostate tumor growth and metastasis, and perhaps other *RB1-*deficient malignancies, with ferroptosis induction.

## Results

### RB1 disruptions sensitize cancer cells to ferroptosis in cancer cell types across various histological origins.

**Figure 1:**
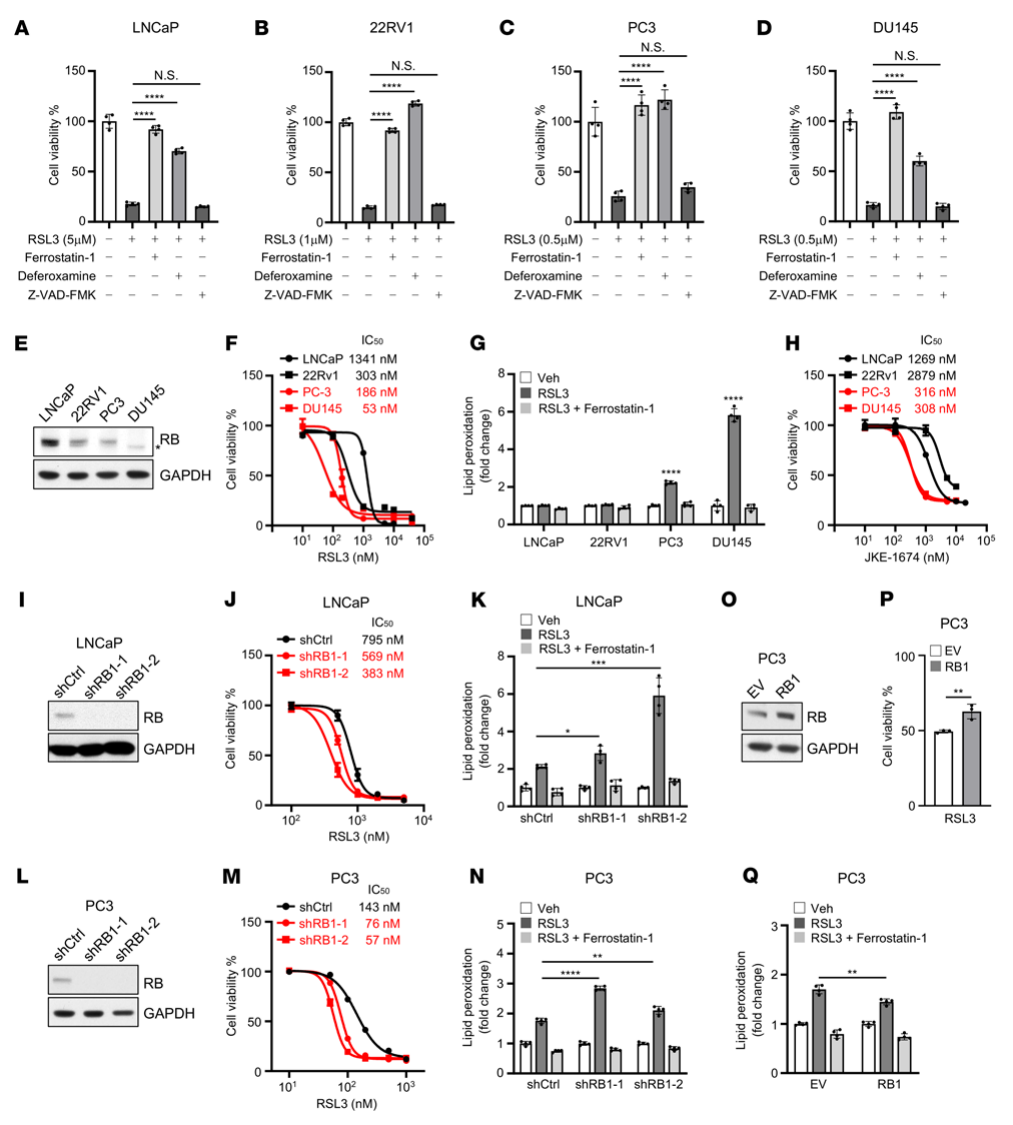
*RB1* disruptions sensitize cancer cells to ferroptosis. (**A**–**D**) 24-hour cell viability assay of LNCaP (**A**), 22RV1 (**B**), PC3 (**C**), or DU145 (**D**) cells treated with the indicated dose of RSL3 in the absence or presence of inhibitors of either ferroptosis (ferrostatin-1 and deferoxamine) or apoptosis (Z-VAD-FMK). (**E**) Immunoblot analysis of lysates from various prostate cancer cell lines. * indicates mutant RB in DU145 cells. (**F** and **G**) 24-hour dose-response curves of RSL3 treatment (**F**) and levels of lipid peroxidation after treatment with 500 nM RSL3 in the absence or presence of ferrostatin-1 for 6 hours (**G**) in various prostate cancer cell lines. (**H**) 24-hour dose-response curves of JKE-1674 treatment in various prostate cancer cell lines. (**I**) Immunoblot analysis of lysates from control or RB stable–knockdown LNCaP cells. (**J** and **K**) 24-hour dose-response curves of RSL3 treatment (**J**) and levels of lipid peroxidation after treatment with 2 μM RSL3 in the absence or presence of ferrostatin-1 for 4 hours (**K**) in control or RB stable–knockdown LNCaP cells. (**L**) Immunoblot analysis of lysates from control or RB stable–knockdown PC3 cells. (**M** and **N**) 24-hour dose-response curves of RSL3 treatment (**M**) and levels of lipid peroxidation after treatment with 500 nM RSL3 in the absence or presence of ferrostatin-1 for 6 hours (**N**) in control or RB stable–knockdown PC3 cells. (**O**) Immunoblot analysis of lysates from PC3 cells transfected with empty vector (EV) or RB1 cDNA for 48 hours. (**P** and **Q**) 24-hour cell viability assay (**P**) and levels of lipid peroxidation in the absence or presence of ferrostatin-1 for 24 hours (**Q**) in EV- or RB-overexpressing PC3 cells treated with 200 nM RSL3. In **A**–**D**, **F**, **H**, **J**, **M**, and **P**, cell viability was measured by CellTiter-Glo 3D cell viability assay. In **G**, **K**, **N**, and **Q**, lipid peroxidation was measured by BODIPY 581/591 C11 fluorescent probe. In **A**–**D**, **G**, **K**, **N**, and **Q**, 1-way ANOVA with Tukey’s multiple-comparison test was used to determine significance. In **P**, unpaired 2-tailed *t* test was used to determine significance. *P < 0.05, **P < 0.01, ***P < 0.001, ****P < 0.0001. All data are shown as the mean ± SD from *n* = 3~4 biological replicates.

We sought to determine how RB regulates ferroptosis. To examine the induction of ferroptosis in vitro in cell-based assays, we treated a panel of human prostate cancer cell lines with a GPX4 inhibitor and widely used in vitro ferroptosis inducer, RSL3, in the absence or presence of inhibitors of either ferroptosis (ferrostatin-1 and deferoxamine) or apoptosis (Z-VAD-FMK). As expected, inhibition of GPX4 by RSL3 specifically induced ferroptosis, but not apoptosis, in all of the prostate cancer cell lines tested (Figure 1, A–D). Interestingly, we observed that prostate cancer cells expressing low (PC3) or mutant (DU145) RB (Figure 1E) required lower doses of RSL3 to reach a similar level of cell death (Figure 1, A–D), were more sensitive to RSL3-induced ferroptosis (Figure 1F), and displayed higher levels of associated lipid peroxidation (Figure 1G). Similar ferroptotic responses were also observed when ferroptosis was induced by JKE-1674 (Figure 1H), a recently discovered GPX4 inhibitor with remarkable selectivity in ferroptosis and vastly improved in vivo stability (33), or imidazole ketone erastin (Supplemental Figure 1A; supplemental material available online with this article; https://doi.org/10.1172/JCI166647DS1), a derivative of the cystine transporter inhibitor erastin (34). To establish a causal relationship between RB and ferroptosis, we tested whether modulation of RB expression would affect the sensitivity of prostate cancer cells to ferroptosis. Depletion of RB by shRNAs (Figure 1I) sensitized LNCaP cells to RSL3-induced ferroptosis and associated lipid peroxidation (Figure 1, J and K). Similar results were also obtained in PC3 cells (Figure 1, L–N). Conversely, overexpression of RB in RB-low PC3 cells (Figure 1O) conferred resistance to ferroptosis and associated lipid peroxidation (Figure 1, P and Q). Importantly, we found that depleting RB in *RB1*-intact cancer cell lines from a range of histological origins, including the lung (A549; Supplemental Figure 1B), liver (HepG2; Supplemental Figure 1D), and breast (MCF7; Supplemental Figure 1F), also sensitized them to ferroptosis induction (Supplemental Figure 1, C, E, and G). These results suggest that RB inhibits ferroptosis in multiple cancer cell types across various histological origins.

Given that ferroptosis-sensitive PC3 and DU145 cell lines also lack expression of the AR, a major driver of prostate cancer progression (35), we examined the regulation of ferroptosis by AR signaling. Stable reintroduction of AR into PC3 or DU145 cells did not affect their sensitivity to ferroptosis (Supplemental Figure 2, A–C) and neither did activation of AR by its ligand dihydrotestosterone or suppression of AR by an antiandrogen, Casodex, in androgen-responsive cell lines, LNCaP and C4-2 (Supplemental Figure 2, D and E). Therefore, AR appears to play a negligible role in the regulation of ferroptosis in human prostate cancer cells.

### The RB/E2F pathway regulates ferroptosis by modulating ACSL4 gene expression.

**Figure 2:**
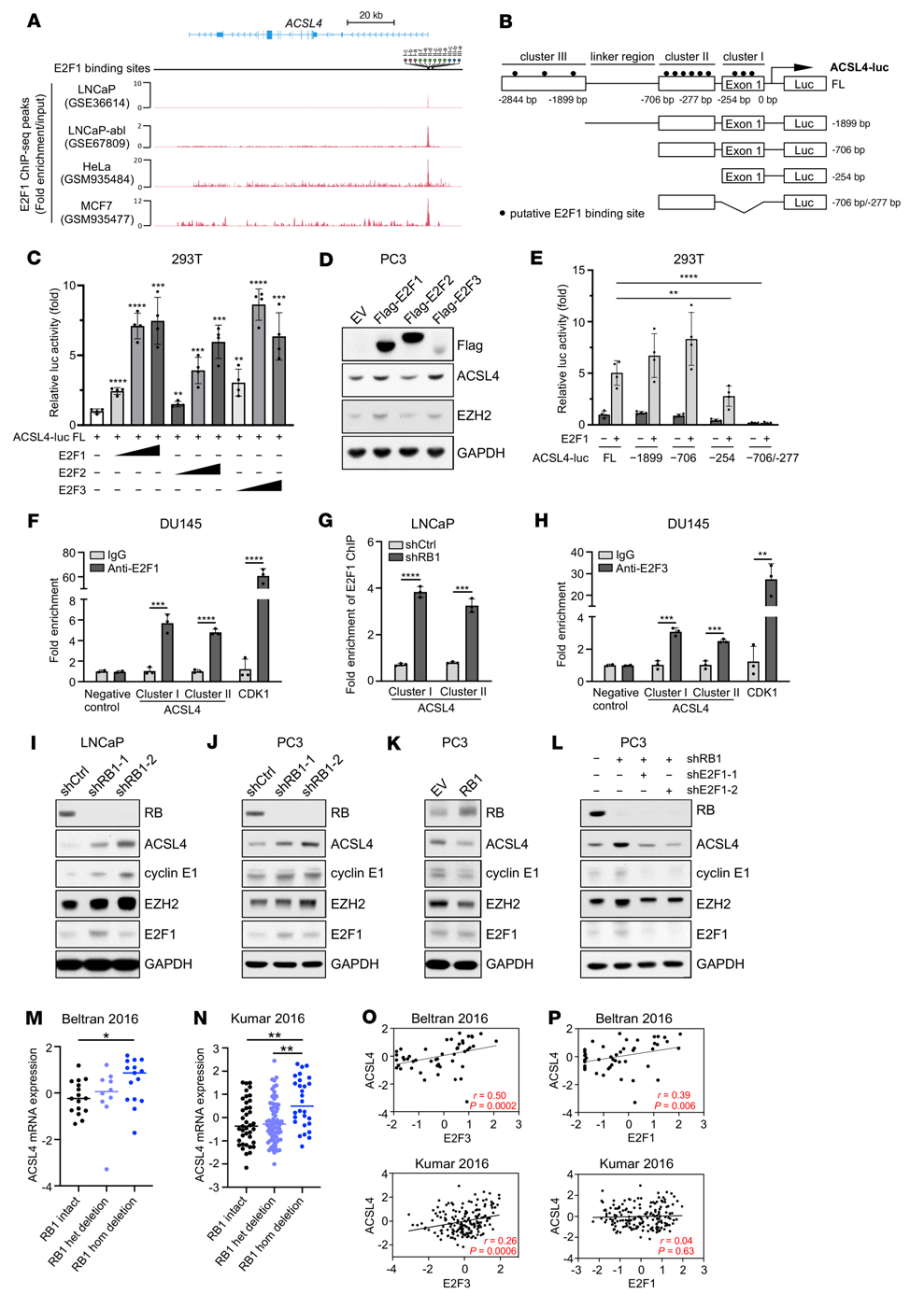
*ACSL4* is a downstream target of the RB/E2F pathway. (**A**) E2F1 ChIP-Seq peaks on the *ACSL4* gene in different cell lines. Twelve putative E2F1 binding sites are indicated at the top of the ChIP-Seq peak. Data were derived from E2F1 ChIP-Seq data sets generated from prostate cancer cell lines (GSE36614 and GSE67809) and HeLa (GSM935484) and MCF7 (GSM935477) cell lines. Note that ChIP-Seq traces show fold enrichment over input for E2F1 binding to *ACSL4* in all cell lines. (**B**) Schematic map of E2F1 binding sites on the 3 kb human *ACSL4* promoter and its full-length and 4 progressive deletion mutant luciferase constructs. (**C**) Luciferase reporter assay of full-length *ACSL4* promoter in the absence or presence of E2F1, E2F2, or E2F3. (**D**) Immunoblot analysis of cell lysates from RB stable–knockdown PC3 cells transfected with EV, E2F1, E2F2, or E2F3 cDNA for 48 hours. (**E**) Luciferase reporter assay of *ACSL4* 3 kb full-length promoter and its progressive deletion mutants in the absence or presence of E2F1. (**F**) ChIP-qPCR of E2F1 binding to the linker region (negative control), cluster I, and cluster II on the promoter of *ACSL4* and *CDK1* loci (positive control) in DU145 cells. (**G**) ChIP-qPCR of E2F1 binding to cluster I and II on the promoter of *ACSL4* in control or RB stable–knockdown LNCaP cells. (**H**) ChIP-qPCR of E2F3 binding to the linker region (negative control), cluster I, and cluster II on the promoter of *ACSL4* and *CDK1* loci (positive control) in DU145 cells. (**I** and **J**) Immunoblot analysis of cell lysates from control or RB stable–knockdown LNCaP (**I**) or PC3 (**J**) cells. (**K**) Immunoblot analysis of cell lysates from PC3 cells transfected with EV or RB1 cDNA for 48 hours. (**L**) Immunoblot analysis of cell lysates from PC3 cells with RB stable–knockdown or combined with E2F1 knockdown. (**M**–**P**) Analysis of correlation between the mRNA levels of *ACSL4* and *RB1* copy number alterations (**M** and **N**) or the mRNA levels of *E2F3* (**O**) or *E2F1* (**P**) in the Beltran et al. (40) or Kumar et al. (41) data set. In **C** and **E**, 1-way ANOVA with Tukey’s multiple-comparison test was used to determine significance. In **F**, **G**, and **H**, unpaired 2-tailed *t* test was used to determine significance. In **M** and **N**, Kruskal-Wallis with Dunn’s multiple-comparison test was used to determine significance. In **O** and **P**, Spearman’s correlation coefficient was used to examine the correlation (*r*, correlation coefficient). *P < 0.05, **P < 0.01, ***P < 0.001, ****P < 0.0001. Bar graphs in **C**, **E**–**H**, are mean ± SD from *n* = 3~4 biological replicates.

The canonical function of RB is to repress E2F-dependent transcription, although E2F-independent functions of RB have recently been proposed (36). To determine the mechanisms underlying RB-regulated ferroptosis, putative E2F1 target genes known as ferroptosis regulators were selected from E2F1 ChIP-Seq data sets generated from prostate cancer cell lines (GEO GSE36614 and GSE67809) and from HeLa (ENCODE GSM935484) and MCF7 (ENCODE GSM935477) cell lines. Among these genes, *ACSL4*, a proferroptotic gene (19–21) with a promoter that was bound by E2F1 in all the ChIP-Seq data sets (Figure 2A), harbored 3 distinct E2F1-binding clusters on its promoter (Supplemental Figure 3A and Figure 2, A and B), as predicted by the JASPAR database of transcription factor binding profiles (37) and ChIP-Seq peaks. The 3 E2F1 binding clusters on the *ACSL4* promoter comprised 3, 6, and 3 putative E2F1 binding sites, respectively, connected by an approximately 1 kb linker region (Supplemental Figure 3A and Figure 2, A and B). The luciferase reporter assay showed that E2F1 and its closely related family members, E2F2 or E2F3, individually led to a largely similar dose-dependent increase in approximately 3 kb *ACSL4* full-length promoter activity (Figure 2C), while overexpression of E2F1 or E2F3, but not E2F2, induced ACSL4 expression in RB-knockdown PC3 cells (Figure 2D). Given that ACSL4 was not upregulated upon E2F2 overexpression, further investigation of the regulation of ACSL4 by E2F family members focused on E2F1 and E2F3. To determine which E2F1 binding cluster contributes to the regulation of the *ACSL4* promoter by E2F1, we generated progressive deletion mutants of the *ACSL4* promoter luciferase reporter (Figure 2B) and found that, compared with the full-length *ACSL4* promoter, deletion of cluster III or the linker region had no significant effect on E2F1-dependent *ACSL4* promoter activity, while deletion of cluster II and I led to approximately 50% and approximately 100% reductions in E2F1-dependent *ACSL4* promoter activity, respectively (Figure 2E). These results suggest that E2F1 transactivates *ACSL4* promoter activity primarily through its binding sites on cluster I and II. Furthermore, luciferase reporter assays using 9 mutants of the *ACSL4* promoter in which putative E2F1 binding sites were mutated one at a time demonstrated that 8 E2F1 binding sites on cluster I and II were required for E2F1-dependent full activation of *ACSL4* promoter activity (Supplemental Figure 3, B and C). In line with in vitro reporter assays, ChIP analysis confirmed the binding of E2F1 to cluster I and II regions, with the linker region on the promoter of *ACSL4* and E2F1 target gene *CDK1* serving as negative and positive controls, respectively (Figure 2F). Using a publicly available RB ChIP-Seq data set generated from C4-2 and VCaP cells (38), we also observed RB binding to the ACSL4 promoter at E2F1-occupied binding sites (Supplemental Figure 3D), suggesting that an RB-E2F1 transcription repressor complex controls ACSL4 expression. Notably, knockdown of RB resulted in significantly increased E2F1 binding to the *ACSL4* promoter (Figure 2G). Consistent with this observation, in two E2F1 ChIP-Seq data sets generated from isogenic *RB1*-proficient and -deficient prostate cancer cell models, E2F1 binding to the ACSL4 promoter was enhanced after RB depletion (Supplemental Figure 3E). Additionally, as in the case of E2F1, E2F3 bound to cluster I and II regions on the promoter of *ACSL4* to a similar degree (Figure 2H). In line with these findings, low levels of RB protein in PC3 cells or functionally inactivated RB in DU145 cells correlated with increased mRNA and protein expression of ACSL4 (Figure 1E and Supplemental Figure 3, F and G), while depletion of RB led to upregulation of ACSL4 and E2F-targeted genes in cancer cell lines from a range of histological origins, including the prostate (LNCaP and PC3; Figure 2, I and J), lung (A549; Supplemental Figure 3H), liver (HepG2; Supplemental Figure 3I), and breast (MCF7; Supplemental Figure 3J), whereas overexpression of RB in RB-low PC3 cells resulted in downregulation of ACSL4 and E2F-target genes (Figure 2K). Critically, knockdown of E2F1 reversed the RB-depletion–induced upregulation of ACSL4 and E2F-target genes (Figure 2L). These findings support the notion that *ACSL4* is a downstream target of the RB/E2F pathway.

To assess the pathophysiological relevance of the RB/E2F/ACSL4 axis in human prostate cancer, we utilized 4 publicly available data sets of metastatic castration-resistant prostate cancer from cBioportal (39) to determine whether *RB1* loss/E2F activation is associated with the expression of the ferroptosis regulator ACSL4. We found that homozygous loss of *RB1* correlated with higher expression of *ACSL4* in the Beltran et al. (40), the Kumar et al. (41), and the Robinson et al. (42) data sets but not in the Abida et al. (14) data set (Figure 2, M and N, and Supplemental Figure 4, A and B), while the expression of *E2F3*, and to a much lesser degree *E2F1*, positively correlated with that of *ACSL4* (Figure 2, O and P, and Supplemental Figure 4, C and D). These findings support the relevance of an aberrant RB/E2F/ACSL4 axis in human prostate cancer.

Given that RB-related proteins, RBL1 and RBL2, can functionally compensate for loss of RB in some settings (43), we investigated whether a similar RBL1/RBL2/E2F/ACSL4 axis exists in human prostate cancer. We found that, similar to RB, depletion of RBL1 or RBL2 led to upregulation of ACSL4 and E2F-targeted genes and sensitized prostate cancer cells to ferroptosis induction (Supplemental Figure 4, E–H). However, homozygous loss of *RBL1* or *RBL2* was rarely observed in metastatic castration-resistant prostate cancer samples from various data sets, and no positive association was found between *ACSL4* expression and inactivation of *RBL1* or *RBL2* (Supplemental Figure 4, I and J). Our data are consistent with those of previous reports that the tumor suppressor properties of the *RB1* gene are significantly stronger than those of *RBL1* and *RBL2* and that among RB family members only *RB1* inactivation is commonly found in human cancer (2, 3).

### ACSL4 is critical for RB-regulated ferroptosis.

**Figure 3:**
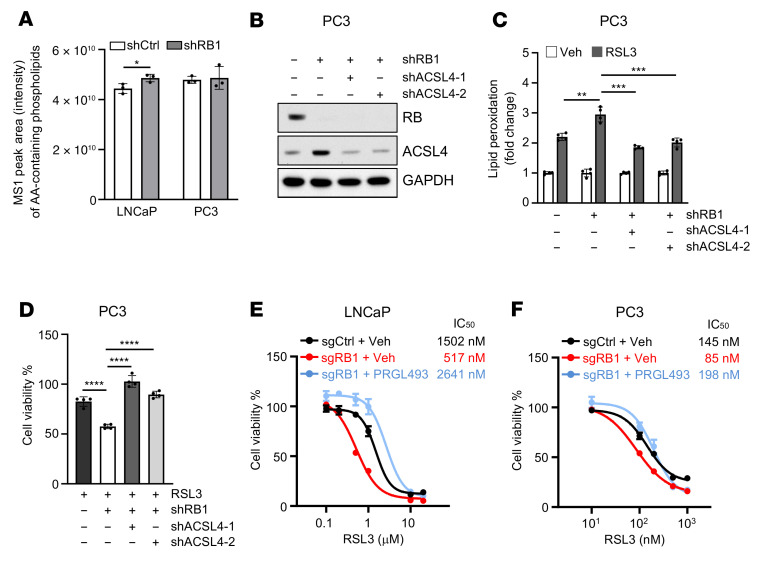
ACSL4 is critical to RB-regulated ferroptosis. (**A**) Bar graph showing the relative MS1 peak area intensity of all the identifiable AA-containing phospholipids in control or RB stable–knockdown LNCaP or PC3 cells. (**B**) Immunoblot analysis of cell lysates from of control or RB stable–knockdown PC3 cells in the absence or presence of ASCL4 stable knockdown. (**C** and **D**) Levels of lipid peroxidation after 6 hours of 1 μM RSL3 treatment (**C**) or cell viability assay after 24 hours of 50 nM RSL3 treatment (**D**) in control or RB stable–knockdown PC3 cells in the absence or presence of ACSL4 stable knockdown. (**E** and **F**) 24-hour dose-response curves of RSL3 treatment in control or *RB1* stable–knockout LNCaP (**E**) or PC3 (**F**) cells pretreated with vehicle or 20 μM PRGL493 for 24 hours. In **A**, unpaired 2-tailed *t* test was used to determine significance. In **C** and **D**, 1-way ANOVA with Tukey’s multiple-comparison test was used to determine significance. *P < 0.05, **P < 0.01, ***P < 0.001, ****P < 0.0001. All data are shown as the mean ± SD from *n* = 3~4 biological replicates.

The induction of ferroptosis relies on the peroxidation of PL-PUFAs (44). ACSL4 facilitates the incorporation of long-chain PUFAs into phospholipids with a significantly greater preference for AA in vitro and in vivo (45, 46) and dictates cellular sensitivity to ferroptosis (19–21). To confirm the involvement of ACSL4 in *RB1* loss–induced sensitization to ferroptosis, we performed a global lipidomic analysis in control and RB-knockdown LNCaP and PC3 cells by using untargeted high-resolution liquid chromatography–tandem mass spectrometry (LC-MS/MS) (47, 48). These analyses identified 1,654 lipid ions in human prostate cancer cell lines (Supplemental Tables 1 and 2), which belonged to 39 classes of lipids (Supplemental Table 3), results largely similar to our previous findings in the mouse prostate (47). We found that AA-containing phospholipids had significantly greater abundance in RB-knockdown LNCaP cells than in control LNCaP cells (Figure 3A and Supplemental Table 4). A trend of increasing levels of AA-containing phospholipids was also observed in RB-knockdown PC3 cells compared with control PC3 cells (Figure 3A and Supplemental Table 4), although this was not statistically significant, possibly due to high basal levels of ACSL4 expression and AA-containing phospholipids in PC3 cells (Supplemental Figure 3, F and G, and Figure 3A). These data indicate that depletion of RB is accompanied by an enrichment of ACSL4-dependent AA-containing phospholipids. Notably, in RB-depletion cells knockdown of ACSL4 led to lower levels of RSL3-induced lipid peroxidation (Figure 3, B and C), which reduced their sensitivity to ferroptosis to levels exhibited by control cells (Figure 3D). In line with these results, inhibition of ACSL4 by PRGL493, a newly discovered ACSL4 inhibitor (49), blunted the sensitivity of *RB1*-knockout cells to ferroptosis (Figure 3, E and F). Collectively, these results suggest that ACSL4 is a key downstream mediator of *RB1* loss–induced sensitization to ferroptosis.

### Induction of ferroptosis suppresses RB1-deficient prostate tumor growth and metastasis.

**Figure 4:**
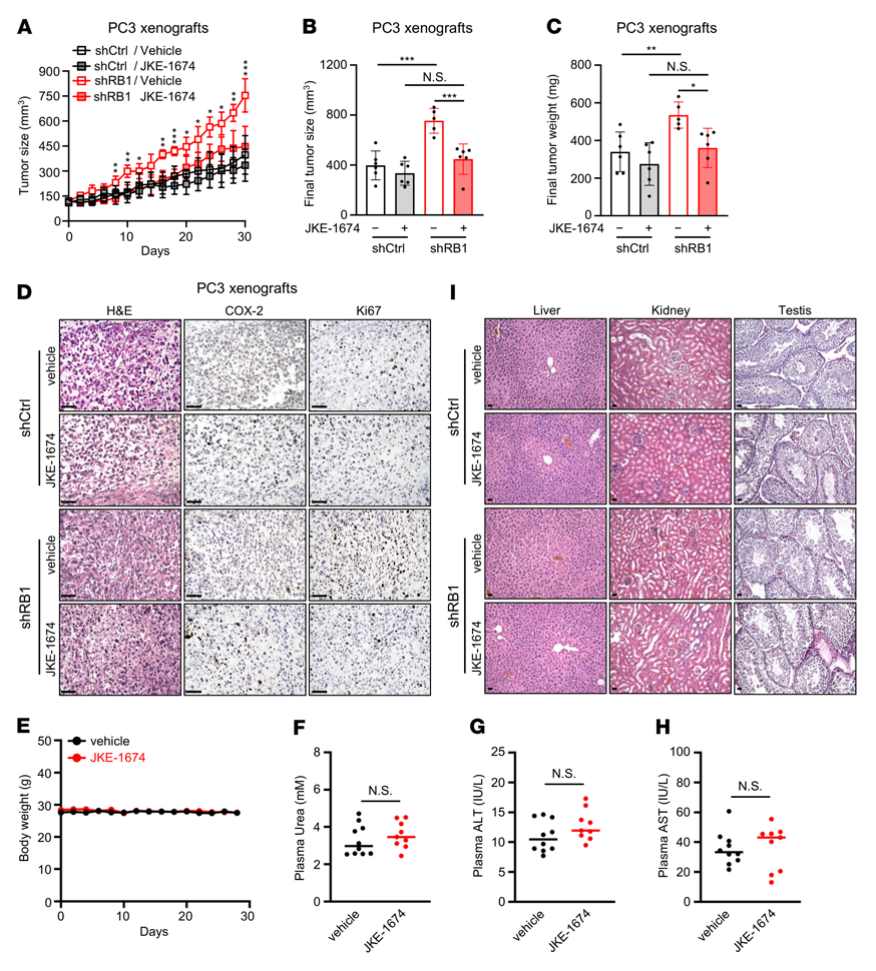
Induction of ferroptosis suppresses the growth of RB-knockdown PC3 xenografts. (**A**–**C**) Tumor volume over the course of treatment (**A**), final tumor volume (**B**), or final tumor weight (**C**) of control or RB stable–knockdown PC3 xenografts after 4-week treatment with vehicle or JKE-1674. *n* = 5~6 mice per group. (**D**) H&E and IHC staining of control or RB stable–knockdown PC3 xenografts after 4-week treatment with vehicle or JKE-1674. Scale bars: 25 μm. (**E**–**I**) Body weight (**E**), the levels of plasma urea (**F**), alanine transaminase (ALT) (**G**), aspartate aminotransferase (AST) (**H**), and H&E staining of liver, kidney, and testis tissues (**I**) of nude mice after implantation of control or RB stable–knockdown PC3 xenografts and treatment with vehicle or JKE-1674 for 4 weeks. Scale bars: 25 μm. JKE-1674 was administered orally at a dose of 25 mg/kg body weight every other day. In **A**–**C**, 2-way ANOVA with Tukey’s multiple-comparison test was used to determine significance. In **F**–**H**, unpaired 2-tailed *t* test was used to determine significance. In **A**, the comparison between vehicle- and JKE-1674– treated RB stable–knockdown PC3 xenografts is shown. *P < 0.05, **P < 0.01, ***P < 0.001. ****P < 0.0001. Data are shown as the mean ± SD.

To test whether ferroptosis could represent a cancer vulnerability elicited by *RB1* deficiency, we carried out in vivo preclinical studies of ferroptosis-inducing therapy in two distinct but complementary tumor model systems. JKE-1674 was utilized in these studies, because RSL3, used in the in vitro studies shown in Figures 1–3, is not generally suitable for in vivo use owing to its poor solubility and unfavorable absorption and metabolization (23). Moreover, JKE-1674 induced ferroptosis as potently as RSL3 in various human cancer cells (33), including prostate cancer cells (Figure 1, F and H). Notably, the IC_50_ of JKE-1674 or RSL3 was markedly higher in the nontumorigenic and normal-like prostate epithelial cell lines, RWPE-1 and BPH-1 (Supplemental Figure 5, A and B, ranging from 9~36 μM for JKE-1674 and 2~4 μM for RSL3), than in prostate cancer cell lines (Figure 1, F and H, ranging from 0.3~2.9 μM for JKE-1674 and 0.05~1.3 μM for RSL3), suggesting a high specificity of ferroptosis inducers for prostate cancer cells. We first tested the in vivo efficacy of JKE-1674 against prostate cancer growth using cell line–derived xenograft models. Treatment with JKE-1674 for 4 weeks significantly inhibited the volume and weight of RB-knockdown PC3 xenografts by 40.6% and 30.3%, respectively (Figure 4, A–C), but had a minor and insignificant antitumor effect in control PC-3 xenografts (Figure 4, A–C), suggesting that *RB1* depletion markedly sensitizes prostate tumors to JKE-1674 treatment. Upon JKE-1674 treatment, RB-depleted tumors displayed higher expression of the ferroptosis biomarkers, COX-2 (Figure 4D) (28) and 4-hydroxynonenal (Supplemental Figure 5C) (24), supporting JKE-1674 as a highly selective ferroptosis inducer. Importantly, JKE-1674 showed no obvious toxicity in mice, as its administration did not alter body weight (Figure 4E), plasma urea (Figure 4F), alanine transaminase (ALT) (Figure 4G), aspartate aminotransferase (AST) (Figure 4H), or the histology of major organs (Figure 4I). These data indicate the feasibility and efficacy of ferroptosis induction against *RB1*-deficient prostate tumorigenesis.

**Figure 5:**
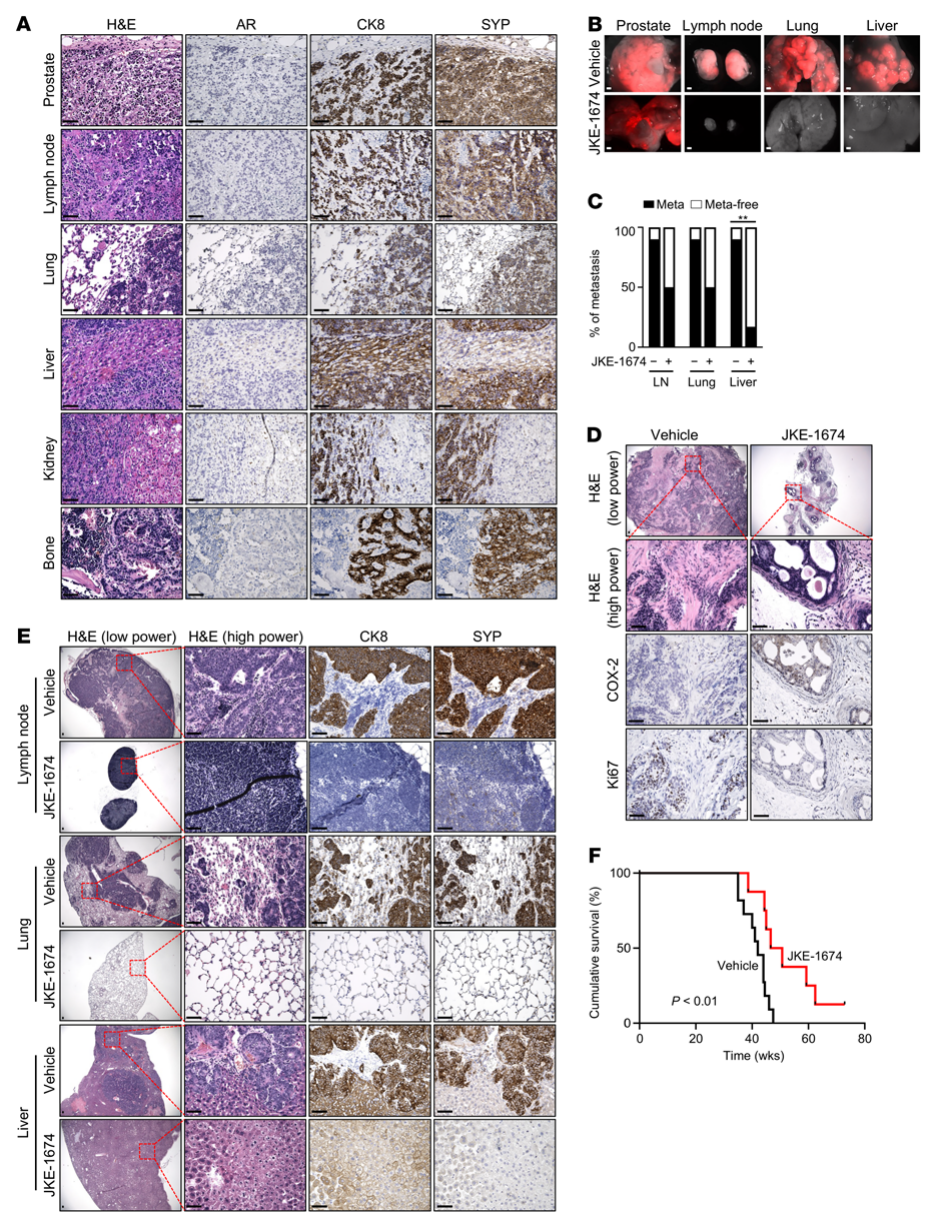
Induction of ferroptosis suppresses prostate tumor growth and metastasis in PPR-RFP mice. (**A**) H&E and IHC staining of end-stage prostate tumors and distant metastases from a representative PPR-RFP mouse. Scale bars: 25 μm. (**B**) Whole-organ fluorescence imaging of the prostate, lymph node, lung, and liver from representative PPR-RFP mice after 6-week treatment with vehicle or JKE-1674. Scale bars: 2 mm. (**C**) The incidence of metastasis in a cohort of PPR-RFP mice after 6-week treatment with vehicle or JKE-1674. *n* = 10 for vehicle-treated mice; *n* = 6 for JKE-1674–treated mice. (**D** and **E**) H&E and IHC staining of prostate tumors (**D**) and lymph node, lung, and liver (**E**) from representative PPR-RFP mice after 6-week treatment with vehicle or JKE-1674. Scale bars: 25 μm. (**F**) Cumulative survival analysis of PPR-RFP mice treated with vehicle or JKE-1674. *n* = 11 for vehicle-treated mice; *n* = 8 for JKE-1674–treated mice. JKE-1674 was administered orally at the dose of 25 mg/kg body weight every other day. In **C**, Fisher’s exact test (2 tailed) was used to determine significance. In **F**, log-rank test was used to determine significance. **P < 0.01.

To further validate the therapeutic potential of ferroptosis induction against *RB1*-deficient prostate cancer, we performed in vivo preclinical studies of JKE-1674 in prostate epithelium-specific *Pten*/*Rb1* double-knockout mice, with prostate tumors that were lineage traced by a bright and stable RFP (subsequently referred to as PPR-RFP mice). *PTEN* and *RB1* are frequently codeleted in late-stage human prostate cancer, including castration-resistant prostate adenocarcinoma and treatment-emergent neuroendocrine prostate cancer (13, 14, 40, 50, 51). While complete inactivation of *Pten* alone in mouse prostate leads to indolent prostate adenocarcinoma that rarely metastasizes to distant organs (6, 47), *Rb1* loss drives lineage plasticity, metastasis, and lethality of prostate tumors initiated by *Pten* loss (6). In findings similar to the previous report (6), we found that PPR-RFP mice developed lethal prostate cancer that metastasized to the lymph node, lung, and liver at 90% penetrance and to the bone (37.5%) and kidney (25%) at moderate-to-low penetrance (Figure 5A and Supplemental Figure 6A). These mice invariably succumbed to their metastatic disease within 46 weeks (Supplemental Figure 6B). The end-stage PPR-RFP primary prostate tumors were poorly differentiated, with little or no glandular structure, and displayed strong coexpression of the luminal epithelial marker cytokeratin 8 (CK8) and the neuroendocrine marker synaptophysin (SYP), along with AR-negative tumors as their primary tumor components (Figure 5A). All metastases resembled primary prostate tumors and showed high levels of CK8 and SYP coexpression along with nearly undetectable expression of nuclear AR (Figure 5A). We enrolled PPR-RFP mice at 7.5 months of age, by which time circulating PPR tumor cells marked by RFP had begun to emerge in peripheral blood (Supplemental Figure 6C). Consistent with the results obtained in our xenograft tumor models, treatment with JKE-1674 for 6 weeks inhibited *Rb1* loss–driven tumorigenesis in terms of both primary tumor growth and distant metastasis to the lymph node, lung, and liver, as indicated by whole-organ fluorescence imaging (Figure 5B and Supplemental Figure 6D) and comparison of the incidence of metastasis between vehicle- and JKE-1674–treated PPR-RFP mice (Figure 5C). Histopathological analyses corroborated that JKE-1674–treated PPR-RFP prostate tumors, compared with vehicle-treated tumors, were largely well-differentiated and displayed higher expression of COX-2, a ferroptosis biomarker (28), along with a concomitant decrease in the frequency of mitotic cells positive for Ki67 staining (Figure 5D and Supplemental Figure 6E), suggesting that poorly differentiated and highly proliferative PPR-RFP tumor cells may be more vulnerable to ferroptosis induction. Notably, after JKE-1674 treatment some PPR-RFP mice were free of distant metastasis (Figure 5, B and C, and Supplemental Figure 6D). These mice displayed a normal appearance of lymph node, lung, and liver where histopathological analyses failed to detect prostate tumor cells coexpressing CK8 and SYP (Figure 5E and Supplemental Figure 6F). We next determined whether JKE-1674 treatment affects the survival of PPR-RFP mice. Seven-and-a-half-month-old PPR-RFP mice were treated with vehicle or JKE-1674 every other day until death. We found that JKE-1674 treatment significantly extended median overall survival of PPR-RFP mice from 42 to 49 weeks (Figure 5F), further corroborating the potent antitumor and antimetastatic activity exhibited by ferroptosis against *RB1*-deficient prostate cancer. Taken together, these preclinical data indicate that *RB1*-deficient tumor growth and metastasis are vulnerable to ferroptosis induction.

## Discussion

Our data establish the RB/E2F/ACSL4 axis as a rheostat of ferroptosis that can be exploited for the treatment of *RB1* loss–driven prostate cancer. Ferroptosis has emerged as a cause of cell death associated with both normal and pathological development; it has been highly conserved from protozoa, plants, and fungi to metazoans (22), and it represents a promising approach to treating cancers of various histological origins in a preclinical setting (23–29). Our findings suggest that the therapeutic efficacy of ferroptosis induction would be higher in patients whose cancers lack functional RB. As *RB1* inactivation is a genomic driver of resistance to several targeted therapies, including AR antagonists (5–7), ER antagonists (8, 9), CDK4/6 inhibitors (10), and EGFR tyrosine kinase inhibitors (11) and it predicts poor clinical outcomes across cancer types (52), our study provide a solution to the pressing need for more effective treatment of *RB1-*deficient malignancies. Notably, our data indicate that the ferroptosis inducer JKE-1674 was more selective in inducing ferroptosis in cancer cells compared with normal-like prostate epithelial cells (Figure 1H and Supplemental Figure 5A) and displayed no obvious toxicity in mice (Figure 4, E–I), demonstrating the feasibility of ferroptosis induction as a cancer therapy. As JKE-1674 is a newly generated GPX4 inhibitor, its pharmacokinetics in vivo has yet to be optimized (33). Further preclinical studies are needed to evaluate whether systematic optimization of dose and duration of JKE-1674 will yield improved therapeutic potential in terms of ferroptosis induction.

RB is a pleiotropic tumor suppressor that exerts its functions in an E2F-dependent and -independent manner (36). Our data reveal that *RB1* loss–induced sensitization to ferroptosis is mediated, at least in part, by ACSL4, a genuine E2F target gene. Notably, among activator E2Fs, E2F3 appeared to induce ACSL4 expression to the greatest degree in RB-knockdown PC3 cells (Figure 2D) and displayed a stronger correlation with ACSL4 in human prostate cancer samples than E2F1 (Figure 2, O and P, and Supplemental Figure 4, C and D), suggesting that E2F3 may be the primary E2F member driven by *RB1* loss to regulate ACSL4 expression in prostate cancer. This differs from the regulation of AR by the RB/E2F axis, where E2F1 is the primary activator (53), but is consistent with a recent study in which *Rb1* loss in a murine intestinal model repositioned Myc and E2f3 from an S/G_2_ program essential for normal cell cycles to a G_1_/S program that reengaged ectopic cell cycles (54). Further studies are needed to determine the potential role of the E2F3 cistrome in mediating *RB1* loss–driving prostate cancer progression and therapeutic vulnerabilities. Furthermore, it remains possible that the regulation of ferroptosis by RB is influenced by its other downstream effectors. For example, *RB1* loss leads to an E2F1-dependent increase in the synthesis of glutathione in advanced disease, presumably protecting cancer cells from reactive oxygen species in response to therapeutic intervention, which renders these cells more reliant on glutathione metabolism for survival and sensitizes them to ferroptosis induced by glutathione-depleting agents, such as erastin and buthionine sulfoximine (55). Additionally, unrestrained E2F activity in the absence of functional RB is known to trigger activation of p53 (56, 57), which regulates ferroptosis in a context-dependent manner (58).

In agreement with findings from recent studies showing that ACSL4 facilitates the incorporation of AA into all species of phospholipids in vivo (46), our lipidomic analysis showed that RB-knockdown cells were enriched in ACSL4-dependent AA-containing phospholipids, which renders them more sensitive to ferroptosis. It is worth noting that ACSL4 is upregulated in therapy-resistant prostate and breast cancer (59, 60), in which *RB1* inactivation most commonly occurs (10, 13, 14, 40). Indeed, we observed that homozygous loss of *RB1* correlates with higher expression of ACSL4 in the data sets of metastatic castration-resistant prostate cancer (Figure 2, M and N, and Supplemental Figure 4A). ACSL4 can be targeted by chemical inhibitors such as PRGL493 (49). Given that complete inactivation of *Acsl4* in mice is compatible with life (61), and that targeting ACSL4 has the advantage of blocking the utilization of fatty acids in cancer cells regardless of whether they are synthesized de novo or acquired exogenously (62), it is tempting to speculate that pharmacological inhibition of ACSL4 may represent a particularly appealing and effective approach to treating *RB1* loss–driven prostate cancer.

All together, our findings reveal the regulation of ferroptosis by the RB/E2F/ACSL4 axis and highlight the therapeutic potential of ferroptosis induction in the treatment of *RB1* loss–driven prostate tumor growth and metastasis and perhaps other *RB1-*deficient malignancies.

## Methods

### Murine models.

The PB-*Cre*4–transgenic mice and *Pten*^fl/fl^ mice have been previously described (47). *Pten*^fl/fl^ mice were first crossed with PB-*Cre*4 mice and lineage marked by ROSA-tdTomato (The Jackson Laboratory, stock 007914) reporter mice. The resulting compound mice were then crossed with *Rb1*^fl/fl^ mice (The Jackson Laboratory, stock 026563) to generate conditional double knockout of *Pten* and *Rb1* in the prostate epithelium and lineage marking by ROSA-tdTomato. The 3 genotypes of mice were maintained on a C57BL/6 background.

### Plasmids, reagents, and antibodies.

Glycerol stocks of pLKO.1 shRNA plasmids targeting RB1, RBL1, RBL2, E2F1, and ACSL4 were purchased from Horizon (TRCN0000010418/TRCN0000010419 for RB1; TRCN0000040018/TRCN0000040021 for RBL1; TRCN0000039923/TRCN0000039926 for RBL2; TRCN0000000249/TRCN0000000252 for E2F1; TRCN0000045539/TRCN0000045540 for ACSL4). Rc/CMV RB1 and LentiCRISPR v2 were purchased from Addgene. The sgRNAs targeting RB1 were designed using Broad Institute GPP sgRNA Designer (https://portals.broadinstitute.org/gpp/public/). The sgRNA oligo pairs containing BsmBI compatible ends (Supplemental Table 5) were synthesized from IDT and annealed. Golden gate assembly with the BsmBI enzyme was used to clone the annealed oligos into LentiCRISPR v2 vectors. Human E2F1, E2F2, or E2F3 cDNA was cloned into pCMV-Tag2B vector to generate its expression plasmid, respectively. The putative E2F1 binding sites on human *ACSL4* promoter was predicted with JASPAR 2020 transcription factor binding profile database (http://jaspar.genereg.net/). An approximately 3 kb *ACSL4* full-length promoter containing all 3 E2F1 binding clusters and its 4 progressive deletion mutants were cloned from normal prostate tissue genomic DNA (Origene) using the Phusion High-Fidelity PCR kit (Thermo Fisher Scientific) with specific primer sets (Supplemental Table 6) into pGL3-basic vector (Promega) for luciferase reporter assay. All mutant constructs of *ACSL4*-Luc reporter were generated using a QuikChange II XL Site-Direct Mutagenesis (Agilent Technologies) and confirmed by sequencing. Ampicillin, kanamycin, and puromycin were purchased from Sigma-Aldrich. RSL3, ferrostatin-1, JKE-1674, and PRGL493 were purchased from Cayman Chemical. Imidazole ketone erastin was purchased from Selleckchem. Polyethylenimine (PEI) was purchased from Polysciences. RPMI, DMEM, Opti-MEM–reduced serum media, and FBS were from Thermo Fisher Scientific. Protein G Sepharose 4 Fast Flow beads were purchased from GE Healthcare. The antibodies for Western blotting are listed below: anti-RB1 (4H1), anti-RBL1 (D3P3C), anti-RBL2 (D9T7M), anti-Ezh2 (D2C9), anti-Cyclin E1 (D7T3U), and anti-GAPDH (D16H11) antibodies were purchased from Cell Signaling Technology. Anti-ACSL4 (A305-358A) and anti-E2F1 (A300-765A) antibodies were purchased from Bethyl Laboratories. Anti-AR (N-20) antibody was purchased from Santa Cruz Biotechnology. Anti-PSA (K92110R) antibody was purchased from Meridian Bioscience. For ChIP assays, anti-E2F1 (no. 3742) and normal IgG were purchased from Cell Signaling Technology. Anti-E2F3 (MA5-11319) was purchased from Thermo Fisher Scientific.

### Cell culture and transfection.

LNCaP, PC-3, 22Rv1, DU145, C4-2, A549, HepG2, MCF7, RWPE-1, and 293T cells were purchased from ATCC. BPH-1 cells were purchased Sigma-Aldrich. Cells were checked for mycoplasma using the MycoAlert Mycoplasma Detection Kit (Lonza). The prostate cancer cells were cultured in RPMI medium. A549, HepG2, MCF7 and 293T cells were cultured in DMEM medium. Complete growth media were supplied with 10% FBS, 2 mM glutamine, and 100 U/mL penicillin-streptomycin (Thermo Fisher Scientific). All cells were maintained at 37°C with 5% CO_2_. Transfections were performed using *Trans*IT-X2 (Mirus Bio LLC) according to manufacturer’s instruction. In brief, 1 μg of DNA plasmids were transfected into 1 × 10^5^ cells in a 6-well dish. Cells were recovered into completed media after a 12-hour transfection and then harvested at the indicated times.

### Lentiviral transduction and stable cell line establishment.

To establish stable RB-knockdown or -knockout cells, 293T cells were used for virus packing. Briefly, the pLKO.1/LentiCRISPR v2, psPAX2, pMD2.G plasmids, and PEI (DNA/PEI = 1:4) were mixed in Opti-MEM (Thermo Fisher Scientific) for 15 minutes and transfected into 293T cells. At 48 and 72 hours after transfection, the virus-containing medium was collected and filtered with a 0.45 μm filter. The virus-containing suspension was mixed with fresh culture medium at 1:1 ratio, supplemented with 4 μg/ml polybrene (Santa Cruz Biotechnology), and then applied to the cells. 48 hours after virus infection, cells were selected using puromycin for 48 hours. For CRISPR knockout cell lines, single-cell clones were isolated and validated using Western blotting.

### Cell viability analyses.

To analyze ferroptotic cell death, cell viability was measured using the CellTiter-Glo 3D Cell Viability Assay kit (Promega). Briefly, we plated the cells in white-walled 96-well plates (Falcon) at the density of 1.0 × 10^4^ cells/well and treated the cells with ferroptosis inducers and/or inhibitors for 24 hours. After the treatment, one volume of the CellTiter-Glo 3D reagent was added into each well, mixed on a thermomixer at 750 rpm for 5 minutes, and then incubated at room temperature for another 20 minutes. The luminance signal for a 250-millisecond integration time was measured using a SpectraMax M3 Multi-Mode Microplate Reader (Molecular Devices).

### Lipid peroxidation assay.

The RSL3-induced lipid peroxidation was measured by using the BODIPY 581/591 C11 probe (Thermo Fisher Scientific). Briefly, cells plated in 6-well plates were loaded with 10 μM BODIPY C11 at 37°C for 30 minutes and then treated with RSL3 and ferrostatin-1 for indicated times. After treatment, cells were harvested by trypsinization and resuspended in PBS. The cell suspension was loaded into 96-well black-walled plates and measured the fluorescence signal using a microplate reader. Lipid peroxidation was determined by calculating the ratio of oxidized (Ex/Em = 485:535 nm) to reduced (Ex/Em = 560/591 nm) C11 fluorescence signal.

### RNA extraction, qPCR, and Western blotting.

The total RNA from LNCaP, 22Rv1, PC-3, and DU145 cells was extracted using TRIzol and the PureLink RNA mini kit (Thermo Fisher Scientific) following the manufacturer’s instructions. To eliminate genomic DNA contamination, the PureLink DNase on-column digest kit (Thermo Fisher Scientific) was applied. The total RNA was then transcribed into cDNA using the PrimeScript RT Master Mix (Takara Bio). Triplicate samples were run for qPCR on a ViiA 7 Real-Time PCR System (Thermo Fisher Scientific) using SsoAdvanced Universal SYBR Green Supermix with specific primer sets (Supplemental Table 7). The Ct values were normalized using the level of RPLP0 as a reference gene. For Western blotting, cells were lysed in RIPA buffer (Boston BioProducts) supplemented with Complete Protease Inhibitor Cocktail and Phosphatase Inhibitor Cocktail 2 (Sigma-Aldrich). The protein content of each sample was quantified using the BCA protein assay kit (Thermo Fisher Scientific). Cell lysates were diluted, mixed with 6× Laemmli buffer (Boston BioProducts), and boiled at 95°C for 5 minutes. Denatured proteins were separated on NuPAGE 4%–12% Bis-Tris gels with MOPS buffer SDS running buffer (Thermo Fisher Scientific) and then transferred onto nitrocellulose membranes (GE Healthcare) using the standard wet transfer method. Membranes were blocked with 5% milk at room temperature for 1 hour and then incubated with specific antibodies at 4°C overnight. The HRP-conjugated secondary antibodies and ECL substrate (GE Healthcare) were applied to visualize the bands of specific proteins (see complete unedited blots in the supplemental material).

### ChIP track plot and ChIP qPCR.

The E2F1 and RB ChIP-Seq fold changes over control signals were downloaded from GSE36614 (GEO), GSE67809 (GEO), GSE94958 (GEO), GSE154191 (GEO), ENCSR000EVJ (ENCODE), ENCSR000EWX (ENCODE), and GSE176402 (GEO), respectively. The track plots for ChIP-Seq signals were plotted by trackViewer (v 1.27.13) (63). To confirm the endogenous binding of E2F1 to ACSL4 promoter, ChIP assays were performed as described previously (64). Briefly, cells were fixed in 1% formaldehyde at room temperature for 10 minutes and then harvested and lysed in ChIP lysis buffer (1% SDS, 5 mM EDTA, 50 mM Tris-HCl, pH = 8.1) supplemented with protease inhibitors. Chromatin was fragmented using a sonicator (Branson) and diluted and immunoprecipitated with normal IgG, E2F1, or E2F3 antibody at 4°C overnight. On the next day, the protein G–Sepharose beads were added and incubated at 4°C for 1 hour. The beads were washed sequentially with TSE I (0.1% SDS, 1% Triton X-100, 2 mM EDTA, 20 mM Tris-HCl, pH = 8.1, 150 mM NaCl), TSE II (0.1% SDS, 1% Triton X-100, 2 mM EDTA, 20 mM Tris-HCl, pH = 8.1, 500 mM NaCl), and buffer III (0.25 M LiCl, 1% NP40, 1% deoxycholate, 1 mM EDTA, 10 mM Tris-HCl, pH=8.1) for 10 minutes each and twice with TE buffer. The chromatin was released from the beads using elution buffer (1% SDS with 0.1 M NaHCO_3_) and decrosslinked by heating at 65°C overnight. The QIAquick PCR Purification Kit (Qiagen) was used for DNA fragment purification. To quantify the binding of endogenous E2F1 to ACSL4 promoter, qPCR was performed using SsoAdvanced Universal SYBR Green Supermix with specific primer sets (Supplemental Table 8).

### Copy number analysis.

Data files were downloaded from cBioPortal (39). For the Beltran et al. data set (40), copy number alterations were based on log_2_ copy number values. For cutoff thresholds, values of −0.3 to −0.9 were classified as heterozygous deletions; those lower than −0.9 were classified as homozygous deletions; those 0.3 to 0.9 were classified as 1-copy gains; and those higher than 0.9 were classified as >1-copy gains. For other data sets, “0” reflected no alterations, “–1” was classified as heterozygous deletions, “–2” as homozygous deletions, “1” as 1-copy gains, and “2” as >1-copy gains.

### Histology and IHC.

Mouse tissues were dissected and fixed in 4% paraformaldehyde for histology and IHC analysis. For staining, the tissues were embedded in paraffin according to standard procedures. 5 μm sections were cut and processed for histology or immunostaining. The following primary antibodies were used: 4-hydroxynonenal (Genox, MHN-020P, 1:500), COX-2 (Cell Signaling Technology, D5H5, 1:400), AR [Abcam, EPR1535(2), 1:250], CK8 (Abcam, EP1628Y, 1:250), SYP (Abcam, YE269, 1:400), and Ki67 (Thermo Fisher Scientific, SP6, 1:100). The stained slides were visualized by a bright-field microscope.

### Lipidomics by untargeted high-resolution LC-MS/MS.

The lipidomic analysis was performed as we previously described (47). Briefly, cells in 10 cm dishes at 80%–90% confluency were harvested in PBS, and protein content was measured using the BCA protein assay kit (Thermo Fisher Scientific) for sample normalization. Nonpolar lipids were extracted with MTBE and dried using a SpeedVac Vacuum Concentrator (Thermo Fisher Scientific) with no heat. Lipid samples were resuspended in 35 μL of 50% isopropanol/50% MeOH. 10 μL of samples were injected for reversed-phase (C_18_) LC-MS/MS using a hybrid QExactive Plus Orbitrap mass spectrometer (Thermo Fisher Scientific) coupled to an Agilent 1100 HPLC in DDA mode using positive/negative ion polarity switching (top 8 in both modes). The lipidomics data were analyzed using LipidSearch 4.1.9 software. The software identifies intact lipid molecules based on their molecular weight and fragmentation pattern using an internal library of predicted fragment ions per lipid class, and the spectra were then aligned based on retention time and MS1 peak areas are quantified across sample conditions.

### PC-3 xenografts and in vivo treatment.

1 × 10^6^ shCT or shRB PC-3 cells were mixed with 100 μL Matrigel (Corning) and implanted subcutaneously into the right flanks of 6- to 8-week-old male nude mice (*Foxn1^nu^*, The Jackson Laboratory). The tumor volume was measured using calipers and calculated as *L* × *W*^2^ × 0.52, where *L* (length) stands for the largest tumor diameter and *W* (width) stands for the diameter perpendicular to the length. When tumor volumes were approximately 80–100 mm^3^ in PC3 xenografts or circulating RFP tumor cells had begun to emerge in peripheral blood of PPR-RFP mice (around 7.5 months), vehicle or JKE-1674 (25 mg/kg, dissolved in 10% ethanol and 90% PEG-400, Sigma-Aldrich) were administered orally to mice every other day.

### Statistics.

No statistics was applied to determine sample size. The studies involving mice were randomized. The investigators were not blinded to allocation during experiments and outcome assessment. Statistical analyses were performed using GraphPad Prism software. For comparison of two experimental groups, an unpaired 2-tailed Student’s *t* test was used. For comparison of more than two groups, 1-way or 2-way ANOVA with Tukey’s multiple-comparison test was used. When data from multiple groups were not normally distributed, a Kruskal-Wallis test followed by Dunn’s multiple-comparison test was used. For analysis of categorical data, 2 × 2 contingency tables were constructed, and data sets were compared using Fisher’s exact test. Survival outcomes were evaluated using Kaplan-Meier survivor estimates and log-rank (Mantel-Cox) test. *P* values of less than 0.05 were considered to be statistically significant.

### Study approval.

Mouse studies were approved by the Duke IACUC under protocol A238-18-10.

## Author contributions

MEW, JC, YL, ARB, JW, JO, and MC performed the experiments. MEW and MC conceived and designed the experiments. AJA, QW, LL, YW, JH, and MC supervised the study. MEW and JMA performed lipidomic analyses. JH conducted pathology analyses of mouse tissues. MEW, JO, JMA, and MC analyzed the data. MEW, JO, and MC wrote the manuscript. All authors critically discussed the results and the manuscript.

## Supplementary Material

Supplemental data

Supplemental tables 1-4

## Version 1 03/16/2023

In-Press Preview

## Version 2 05/15/2023

Electronic publication

## References

[1] Horowitz JM (1990). Frequent inactivation of the retinoblastoma anti-oncogene is restricted to a subset of human tumor cells. Proc Natl Acad Sci U S A.

[2] Burkhart DL, Sage J (2008). Cellular mechanisms of tumour suppression by the retinoblastoma gene. Nat Rev Cancer.

[3] Knudsen ES, Knudsen KE (2008). Tailoring to RB: tumour suppressor status and therapeutic response. Nat Rev Cancer.

[4] Tan HL (2014). Rb loss is characteristic of prostatic small cell neuroendocrine carcinoma. Clin Cancer Res.

[5] Sharma A (2007). Retinoblastoma tumor suppressor status is a critical determinant of therapeutic response in prostate cancer cells. Cancer Res.

[6] Ku SY (2017). Rb1 and Trp53 cooperate to suppress prostate cancer lineage plasticity, metastasis, and antiandrogen resistance. Science.

[7] Mu P (2017). SOX2 promotes lineage plasticity and antiandrogen resistance in TP53- and RB1-deficient prostate cancer. Science.

[8] Varma H, Conrad SE (2000). Reversal of an antiestrogen-mediated cell cycle arrest of MCF-7 cells by viral tumor antigens requires the retinoblastoma protein-binding domain. Oncogene.

[9] Bosco EE (2007). The retinoblastoma tumor suppressor modifies the therapeutic response of breast cancer. J Clin Invest.

[10] Li Z (2018). Loss of the FAT1 tumor suppressor promotes resistance to CDK4/6 inhibitors via the hippo pathway. Cancer Cell.

[11] Niederst MJ (2015). RB loss in resistant EGFR mutant lung adenocarcinomas that transform to small-cell lung cancer. Nat Commun.

[12] Walter DM (2019). RB constrains lineage fidelity and multiple stages of tumour progression and metastasis. Nature.

[13] Chen WS (2019). Genomic drivers of poor prognosis and enzalutamide resistance in metastatic castration-resistant prostate cancer. Eur Urol.

[14] Abida W (2019). Genomic correlates of clinical outcome in advanced prostate cancer. Proc Natl Acad Sci U S A.

[15] Brambilla E (1999). Alterations of expression of Rb, p16(INK4A) and cyclin D1 in non-small cell lung carcinoma and their clinical significance. J Pathol.

[16] Cordon-Cardo C (1992). Altered expression of the retinoblastoma gene product: prognostic indicator in bladder cancer. J Natl Cancer Inst.

[17] Herschkowitz JI (2008). The functional loss of the retinoblastoma tumour suppressor is a common event in basal-like and luminal B breast carcinomas. Breast Cancer Res.

[18] Dixon SJ (2012). Ferroptosis: an iron-dependent form of nonapoptotic cell death. Cell.

[19] Doll S (2017). ACSL4 dictates ferroptosis sensitivity by shaping cellular lipid composition. Nat Chem Biol.

[20] Kagan VE (2017). Oxidized arachidonic and adrenic PEs navigate cells to ferroptosis. Nat Chem Biol.

[21] Dixon SJ (2015). Human haploid cell genetics reveals roles for lipid metabolism genes in nonapoptotic cell death. ACS Chem Biol.

[22] Stockwell BR (2022). Ferroptosis turns 10: Emerging mechanisms, physiological functions, and therapeutic applications. Cell.

[23] Lei G (2022). Targeting ferroptosis as a vulnerability in cancer. Nat Rev Cancer.

[24] Mao C (2021). DHODH-mediated ferroptosis defence is a targetable vulnerability in cancer. Nature.

[25] Ghoochani A (2021). Ferroptosis inducers are a novel therapeutic approach for advanced prostate cancer. Cancer Res.

[26] Yi J (2020). Oncogenic activation of PI3K-AKT-mTOR signaling suppresses ferroptosis via SREBP-mediated lipogenesis. Proc Natl Acad Sci U S A.

[27] Badgley MA (2020). Cysteine depletion induces pancreatic tumor ferroptosis in mice. Science.

[28] Zhang Y (2019). Imidazole ketone erastin induces ferroptosis and slows tumor growth in a mouse lymphoma model. Cell Chem Biol.

[29] Wang W (2019). CD8^+^ T cells regulate tumour ferroptosis during cancer immunotherapy. Nature.

[30] Hangauer MJ (2017). Drug-tolerant persister cancer cells are vulnerable to GPX4 inhibition. Nature.

[31] Viswanathan VS (2017). Dependency of a therapy-resistant state of cancer cells on a lipid peroxidase pathway. Nature.

[32] Louandre C (2015). The retinoblastoma (Rb) protein regulates ferroptosis induced by sorafenib in human hepatocellular carcinoma cells. Cancer Lett.

[33] Eaton JK (2020). Selective covalent targeting of GPX4 using masked nitrile-oxide electrophiles. Nat Chem Biol.

[34] Larraufie MH (2015). Incorporation of metabolically stable ketones into a small molecule probe to increase potency and water solubility. Bioorg Med Chem Lett.

[35] Heinlein CA, Chang C (2004). Androgen receptor in prostate cancer. Endocr Rev.

[36] Dick FA (2018). Non-canonical functions of the RB protein in cancer. Nat Rev Cancer.

[37] Fornes O (2020). JASPAR 2020: update of the open-access database of transcription factor binding profiles. Nucleic Acids Res.

[38] Han W (2022). RB1 loss in castration-resistant prostate cancer confers vulnerability to LSD1 inhibition. Oncogene.

[39] Cerami E (2012). The cBio cancer genomics portal: an open platform for exploring multidimensional cancer genomics data. Cancer Discov.

[40] Beltran H (2016). Divergent clonal evolution of castration-resistant neuroendocrine prostate cancer. Nat Med.

[41] Kumar A (2016). Substantial interindividual and limited intraindividual genomic diversity among tumors from men with metastatic prostate cancer. Nat Med.

[42] Robinson D (2015). Integrative clinical genomics of advanced prostate cancer. Cell.

[43] Dannenberg JH (2004). Tissue-specific tumor suppressor activity of retinoblastoma gene homologs p107 and p130. Genes Dev.

[44] Yang WS (2016). Peroxidation of polyunsaturated fatty acids by lipoxygenases drives ferroptosis. Proc Natl Acad Sci U S A.

[45] Kang MJ (1997). A novel arachidonate-preferring acyl-CoA synthetase is present in steroidogenic cells of the rat adrenal, ovary, and testis. Proc Natl Acad Sci U S A.

[46] Killion EA (2018). A role for long-chain acyl-CoA synthetase-4 (ACSL4) in diet-induced phospholipid remodeling and obesity-associated adipocyte dysfunction. Mol Metab.

[47] Chen M (2018). An aberrant SREBP-dependent lipogenic program promotes metastatic prostate cancer. Nat Genet.

[48] Breitkopf SB (2017). A relative quantitative positive/negative ion switching method for untargeted lipidomics via high resolution LC-MS/MS from any biological source. Metabolomics.

[49] Castillo AF (2021). New inhibitor targeting Acyl-CoA synthetase 4 reduces breast and prostate tumor growth, therapeutic resistance and steroidogenesis. Cell Mol Life Sci.

[50] Aggarwal R (2018). Clinical and genomic characterization of treatment-emergent small-cell neuroendocrine prostate cancer: a multi-institutional prospective study. J Clin Oncol.

[51] Quigley DA (2018). Genomic hallmarks and structural variation in metastatic prostate cancer. Cell.

[52] Chen WS (2019). Novel RB1-loss transcriptomic signature is associated with poor clinical outcomes across cancer types. Clin Cancer Res.

[53] Sharma A (2010). The retinoblastoma tumor suppressor controls androgen signaling and human prostate cancer progression. J Clin Invest.

[54] Liu H (2015). Redeployment of Myc and E2f1-3 drives Rb-deficient cell cycles. Nat Cell Biol.

[55] Mandigo AC (2021). RB/E2F1 as a master regulator of cancer cell metabolism in advanced disease. Cancer Discov.

[56] Pan H (1998). Key roles for E2F1 in signaling p53-dependent apoptosis and in cell division within developing tumors. Mol Cell.

[57] Ruiz S (2006). Is the loss of pRb essential for the mouse skin carcinogenesis?. Cell Cycle.

[58] Kang R (2019). The tumor suppressor protein p53 and the ferroptosis network. Free Radic Biol Med.

[59] Wu X (2013). Long chain fatty Acyl-CoA synthetase 4 is a biomarker for and mediator of hormone resistance in human breast cancer. PLoS One.

[60] Wu X (2015). ACSL4 promotes prostate cancer growth, invasion and hormonal resistance. Oncotarget.

[61] Ren H (2019). Generation of Acsl4 gene knockout mouse model by CRISPR/Cas9-mediated genome engineering. Crit Rev Biomed Eng.

[62] Chen M, Huang J (2019). The expanded role of fatty acid metabolism in cancer: new aspects and targets. Precis Clin Med.

[63] Ou J, Zhu LJ (2019). trackViewer: a Bioconductor package for interactive and integrative visualization of multi-omics data. Nat Methods.

[64] Wang Q (2009). Androgen receptor regulates a distinct transcription program in androgen-independent prostate cancer. Cell.

