# Supplementary material for: RB1-deficient prostate tumor growth and metastasis are vulnerable to ferroptosis induction via the E2F/ACSL4 axis
Source: J Clin Invest. 2023 May 15;133(10):e166647. doi: 10.1172/JCI166647 (PMC10178842; doi:10.1172/JCI166647)

Supplemental Data

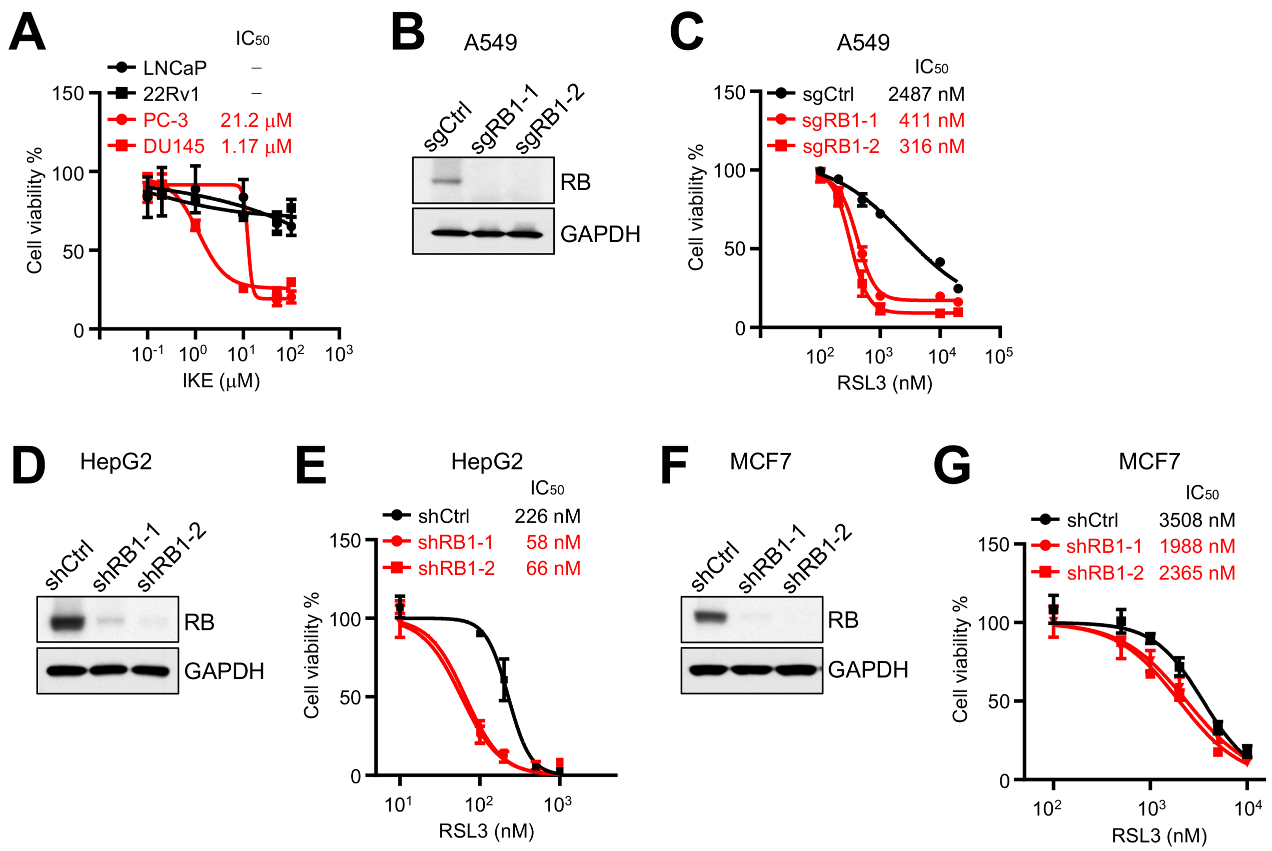

Supplemental Figure 1. *RB1* disruptions sensitize cancer cells to ferroptosis. (**A**) 24-hour dose response curves of IKE treatment in various prostate cancer cell lines. (**B** and **C**) IB analysis (B) and 24-hour dose response curves of RSL3 treatment (C) in control or *RB1* stable knockout A549 cells. (**D** and **E**) IB analysis (D) and 24-hour dose response curves of RSL3 treatment (E) in control or RB stable knockdown HepG2 cells. (**F** and **G**) IB analysis (F) and 24-hour dose response curves of RSL3 treatment (G) in control or RB stable knockdown MCF7 cells. All data are mean ± s.d. from n = 4 biological replicates.

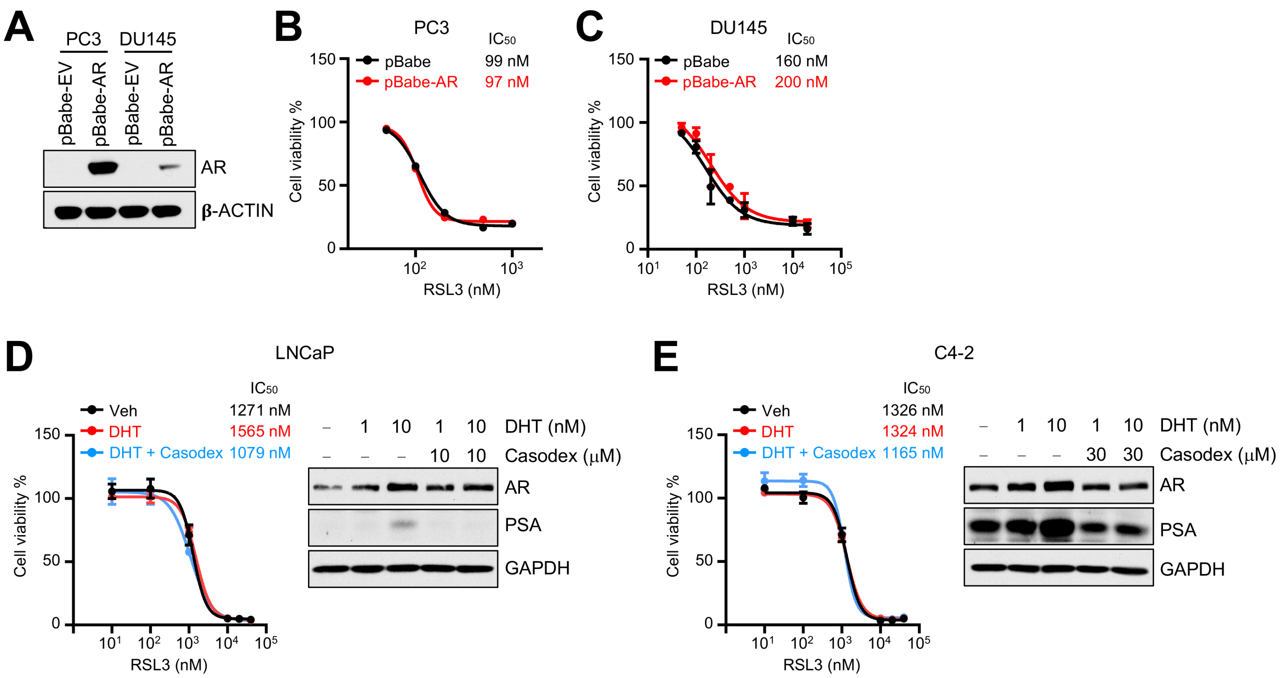

Supplemental Figure 2. The androgen–AR axis plays a negligible role in the regulation of ferroptosis in human prostate cancer cells. (**A**) IB analysis of lysates from control or AR stably overexpressing PC3 or DU145 cells. (**B** and **C**) 24-hour dose response curves of RSL3 treatment in control or AR stably overexpressing PC3 (B) or DU145 (C) cells. (**D** and **E**) 24-hour dose response curves of RSL3 treatment in LNCaP (D) or C4-2 (E) cells pretreated with vehicle or 10 nM DHT in the absence or presence of anti-androgen, Casodex, for 24 hours. IB analysis confirmed the expression of AR and its target gene PSA. All data are mean ± s.d. from n = 4 biological replicates.

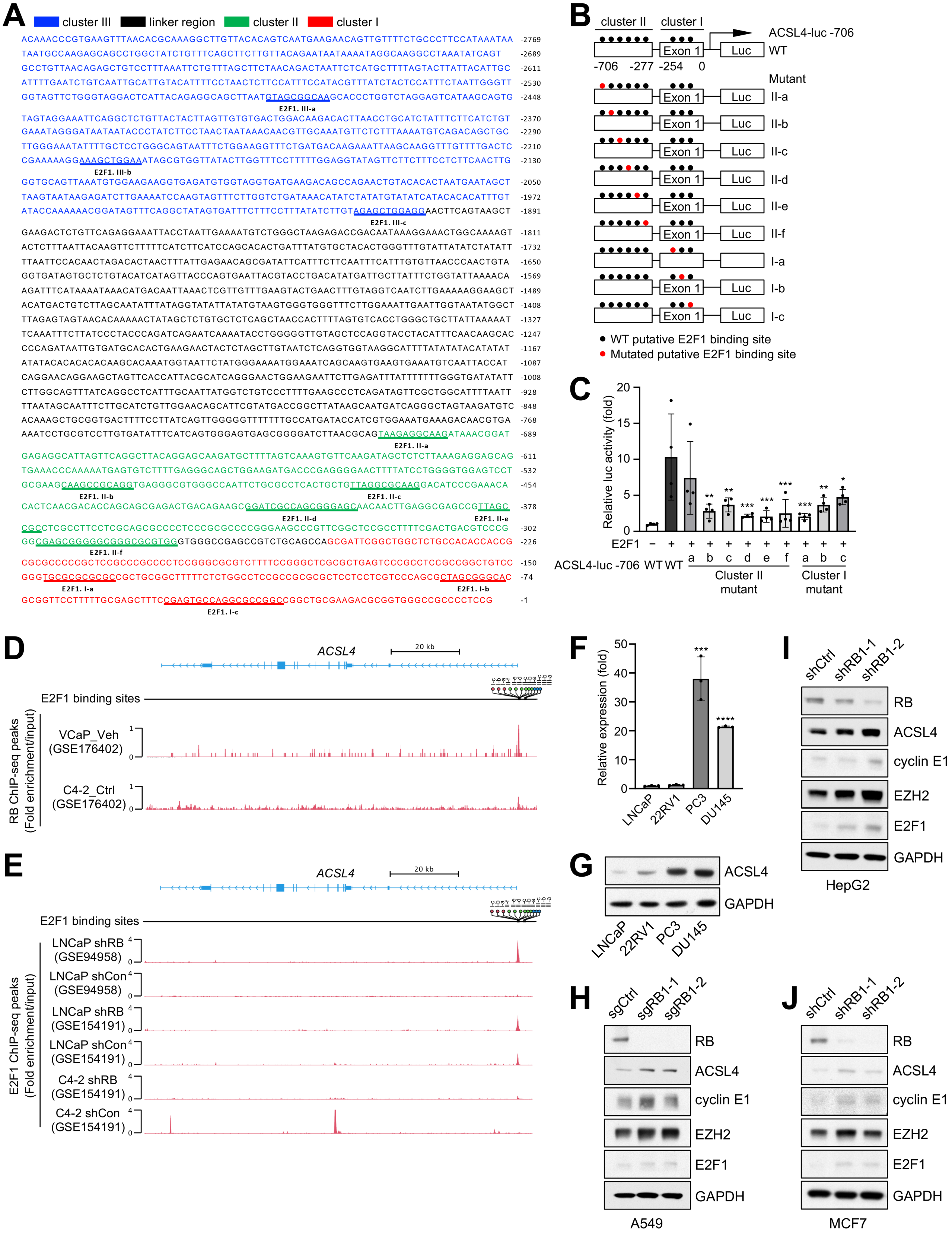

A
cluster III
linker region
cluster II
cluster I
B
cluster II
cluster I
ACSL4-luc -706
WT
Mutant
Exon 1
Luc
II-a
II-b
II-c
II-d
II-e
II-f
I-a
I-b
I-c
WT putative E2F1 binding site
Mutated putative E2F1 binding site
C
Relative luc activity (fold)
E2F1
ACSL4-luc -706
Cluster II mutant
Cluster I mutant
D
ACSL4
20 kb
E2F1 binding sites
RB ChIP-seq peaks (Fold enrichment/input)
VCaP_Veh (GSE176402)
C4-2_Ctrl (GSE176402)
E
E2F1 ChIP-seq peaks (Fold enrichment/input)
LNCaP shRB (GSE94958)
LNCaP shCon (GSE94958)
LNCaP shRB (GSE154191)
LNCaP shCon (GSE154191)
C4-2 shRB (GSE154191)
C4-2 shCon (GSE154191)
F
Relative expression (fold)
LNCaP
22RV1
PC3
DU145
G
ACSL4
GAPDH
H
sgCtrl
sgRB1-1
sgRB1-2
RB
cyclin E1
EZH2
E2F1
A549
I
shCtrl
shRB1-1
shRB1-2
HepG2
J
MCF7

Supplemental Figure 3. ***ACSL4*** **is a novel target gene of the E2F transcription factor family**. (**A**) Nucleotide sequences of the 3-kb human *ACSL4* promoter. Sequences of three E2F1 binding clusters on the *ACSL4* promoter are highlighted in colors and putative E2F1 binding sites are underlined. (**B** and **C**) Schematic map of *ACSL4*-luc-706 construct and its nine mutants in which putative E2F1 binding sites were mutated one at a time (B), and their respective luciferase reporter assays in the absence or presence of E2F1 (C). (**D**) RB ChIP-seq peaks on the *ACSL4* gene in VCaP and C4-2 cells. Twelve putative E2F1 binding sites are indicated at the top of the ChIP-seq peak. Data derived from RB ChIP-seq dataset GSE176402. (**E**) E2F1 ChIP-seq peaks on the *ACSL4* gene in isogenic *RB1*-proficient and -deficient C4-2 and LNCaP cells. Twelve putative E2F1 binding sites are indicated at the top of the ChIP-seq peak. Data derived from E2F1 ChIP-seq datasets, GSE94958 and GSE154191, respectively. (**F** and **G**) Q-PCR (F) and IB analysis (G) of ACSL4 levels in prostate cancer cell lines. (**H** to **J**) IB analysis of lysates from control or RB stably depleted A549 (H), HepG2 (I), or MCF7 (J) cells by CRISPR or shRNA. In C and F, One-way ANOVA with Tukey's multiple-comparison test was used to determine significance. $^{*}P < 0.05$, $^{**}P<0.01$, $^{***}P < 0.001$, $^{****}P < 0.0001$. All data are mean ± s.d. from $n = 4$ biological replicates.

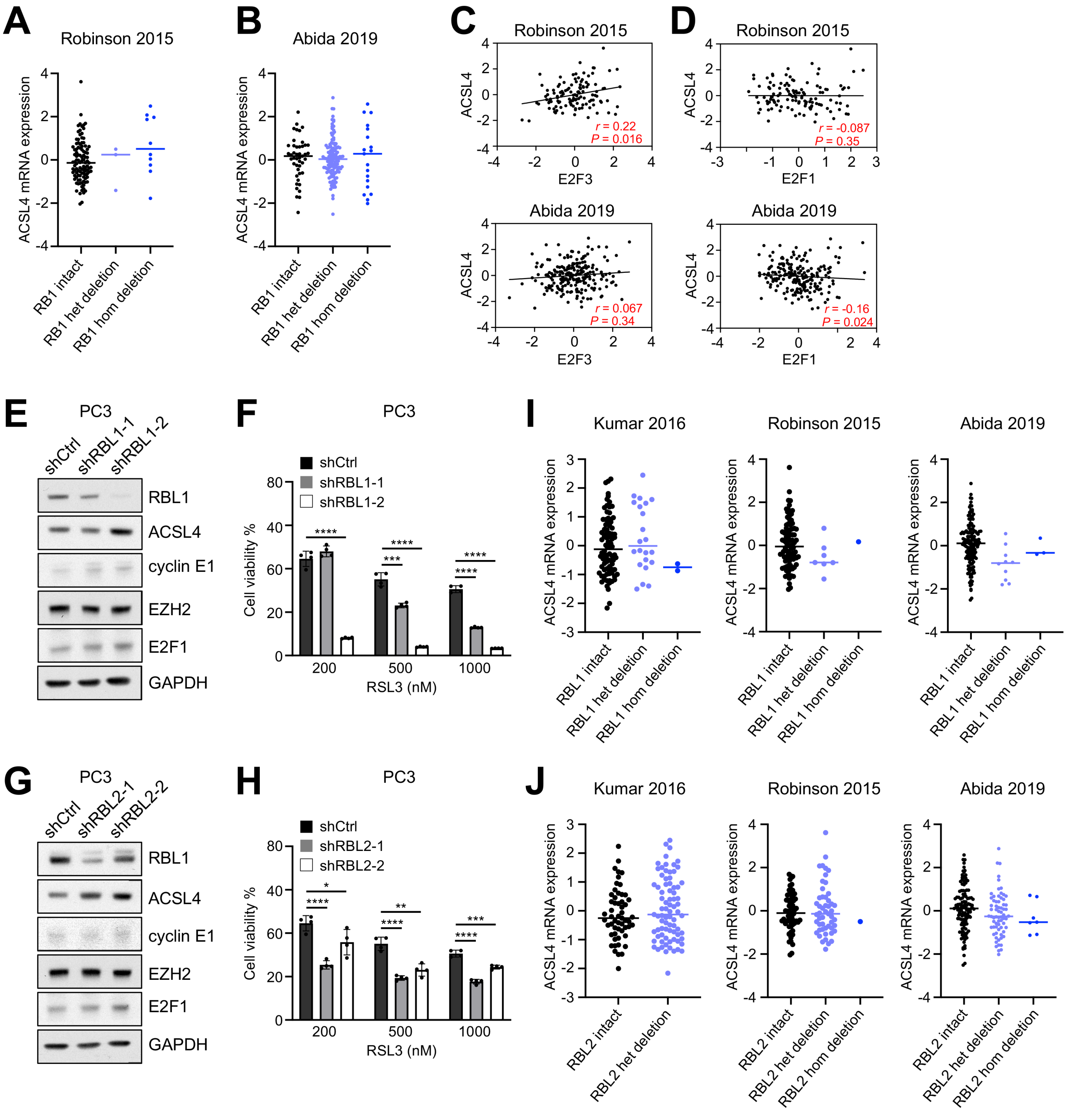

Supplemental Figure 4. The correlation between *RB1* genomic status and ACSL4 in SU2C prostate cancer datasets and the regulation of E2F/ACSL4 by RBL1 or RBL2 and their correlation in human prostate cancer. (**A** to **D**) Analysis of correlation between the mRNA levels of *ACSL4* and *RB1* copy number alterations (A and B) or the mRNA levels of *E2F3* (C) or *E2F1*

(D) in the Robinson et al. (42) or Abida et al. (14) dataset. (**E** and **F**) IB analysis (E) and 24-hour cell viability assay treated with the indicated dose of RSL3 (F) in control or RBL1 stable knockdown PC3 cells. (**G** and **H**) IB analysis (G) and 24-hour cell viability assay treated with the indicated dose of RSL3 (H) in control or RBL2 stable knockdown PC3 cells. (**I** and **J**) Analysis of correlation between the mRNA levels of *ACSL4* and *RBL1* (**I**) *or RBL2* (**J**) copy number alterations in the Kumar et al. (41), the Robinson et al. (42), or the Abida et al. (14) dataset. In A, B, I, and J, Kruskal-Wallis with Dunn's multiple-comparison test was used to determine significance. In C and D, Spearman's correlation coefficient was used to examine the correlation (r, correlation coefficient). Correlation is significant when $P < 0.05$. In F and H, One-way ANOVA with Tukey's multiple-comparison test was used to determine significance. *$P < 0.05$, **$P < 0.01$, ***$P < 0.001$, ****$P < 0.0001$. Bar graphs in F and H, are mean ± s.d. from $n = 4$ biological replicates.

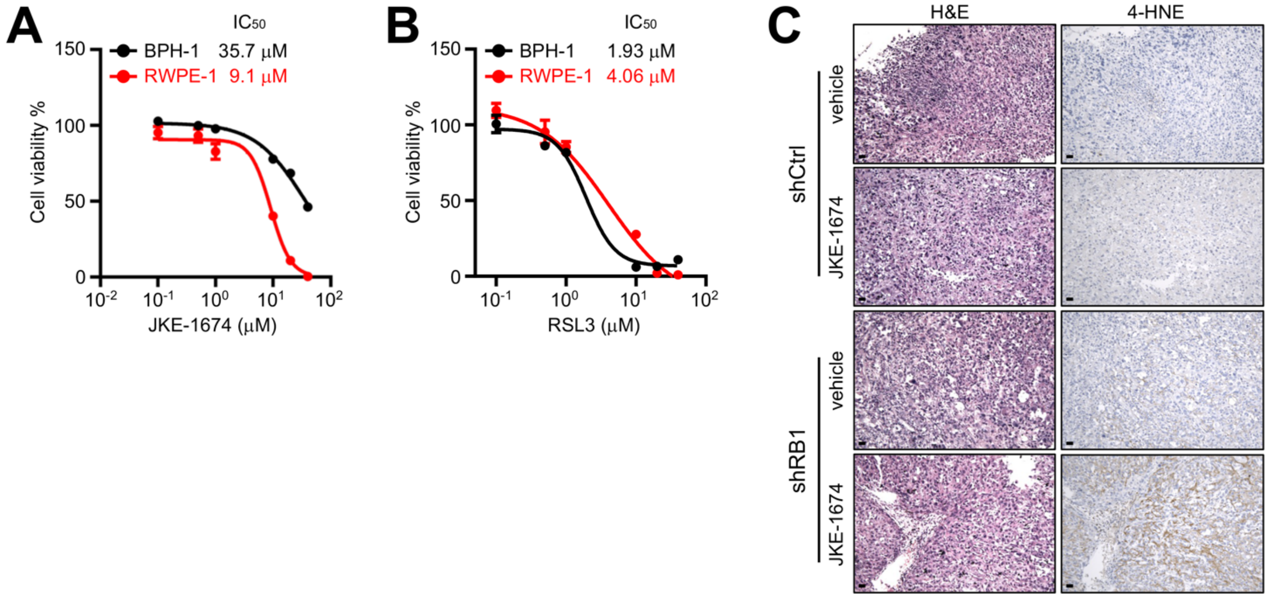

Supplemental Figure 5. Induction of ferroptosis suppresses RB-knockdown PC3 xenograft growth. (**A** and **B**) 24-hour dose response curves of JKE-1674 (A) or RSL3 (B) treatment in RWPE-1 or BPH-1 cells. (**C**) H&E and IHC staining of control or RB stable knockdown PC3 xenografts after four-week treatment with vehicle or JKE-1674. Scale bars, 25μm. JKE-1674 was administered orally at a dose of 25 mg/kg body weight every other day.

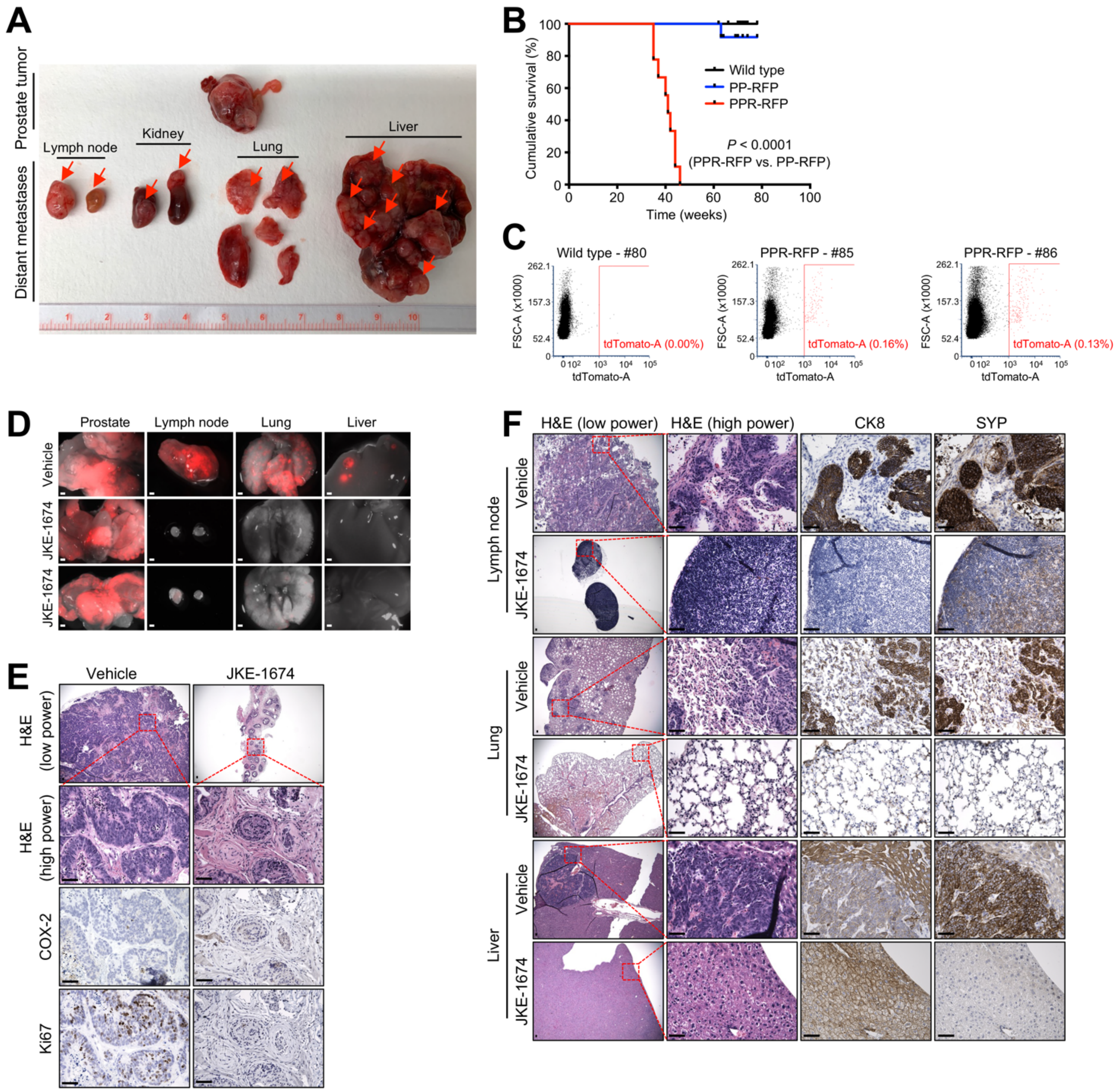

Supplemental Figure 6. Induction of ferroptosis suppresses prostate tumor growth and metastasis in PPR-RFP mice. (**A**) Gross anatomy of primary prostate tumors and distant metastases from a representative PPR-RFP mouse. (**B**) Cumulative survival analysis of wild type, prostate epithelium-specific *Pten* knockout mice lineage traced by RFP (mice referred to as PP-RFP), and PPR-RFP mice. (**C**) Representative FACS plots of RFP expression in peripheral blood from seven and a half-month-old wild type and PPR-RFP mice. (**D** to **F**) Whole-organ fluorescence

imaging of the prostate, lymph node, lung, and liver (D), H&E and IHC staining of prostate tumors (E) or lymph node, lung, and liver (F) from representative PPR-RFP mice after six-week treatment with vehicle or JKE-1674. Scale bars, 2 mm (D) and 25 μm (E and F). JKE-1674 was administered orally at a dose of 25 mg/kg body weight every other day. In B, log-rank test was used to determine significance.

Supplemental Table 5. sgRNA sequences for CRIPSR knock-out experiments.

Primer Name	Sequence (5' to 3')
RB1 sgRNA #1 Forward	CACCGAAGTGAACGACATCTCATCT
RB1 sgRNA #1 Reverse	AAACAGATGAGATGTCGTTCACTTC
RB1 sgRNA #2 Forward	CACCGGGTTCTTTGAGCAACATGGG
RB1 sgRNA #2 Reverse	AAACCCCATGTTGCTCAAAGAACCC

Supplemental Table 6. Primer sets used for ACSL4 promoter cloning.

Primer Name	Sequence (5' to 3')
Full Length Forward	CCCGGGCTCGAGCCCGTGAAGTTTAACACGCA
Full Length Reverse	AATGCCAAGCTTCGGAGGGGCGGCCCACCGCG
Δ Cluster III Forward	CCCGGGCTCGAGAACTTCAGTAAGCTGAAGAC
Δ Cluster III Reverse	AATGCCAAGCTTCGGAGGGGCGGCCCACCGCG
Δ Cluster III/linker Forward	CCCGGGCTCGAGTAAGAGGCAAGATAAACGGA
Δ Cluster III/linker Reverse	AATGCCAAGCTTCGGAGGGGCGGCCCACCGCG
Δ Cluster III/linker/II Forward	CCCGGGCTCGAGGCGATTCGGCTGGCTCTGCC
Δ Cluster III/linker/II Reverse	AATGCCAAGCTTCGGAGGGGCGGCCCACCGCG
Δ Cluster III/linker/I Forward	CCCGGGCTCGAGTAAGAGGCAAGATAAACGGA
Δ Cluster III/linker/I Reverse	AATGCCAAGCTTTGGCTGCAGACGGCTCGG

Supplemental Table 7. Primer sets used for qPCR.

Primer Name	Sequence (5' to 3')
ACSL4 Forward	TGTACTGTACTGAAGCCCACACTT
ACSL4 Reverse	TTCATCTCTTGGACTTTGCTCA
RPLP0 Forward	GCTTCCTGGAGGGTGTCC
RPLP0 Reverse	GGACTCGTTTGTACCCGTTG

Supplemental Table 8. Primer sets used for ChIP qPCR.

Primer Name	Sequence (5' to 3')
ACSL4 promoter linker region Forward	TGTGCTCTCAGCTAACCACTTT
ACSL4 promoter linker region Reverse	GTAGGTACCTGGAGCTACAACC
CDK1 promoter Forward	CGCCCTTTCCTCTTTCTTTC
CDK1 promoter Reverse	ATCGGGTAGCCCGTAGACTT
ACSL4 promoter cluster I Forward	TCCGGGCGCGTCTTTTC
ACSL4 promoter cluster I Reverse	GCAAAAAGGAACCGCGTGC
ACSL4 promoter cluster II Forward	TCACTGCTGTTAGGCGCAA
ACSL4 promoter cluster II Reverse	CGCTTCTGTCAGTCTCGCT

Full unedited gel for Figure 1E

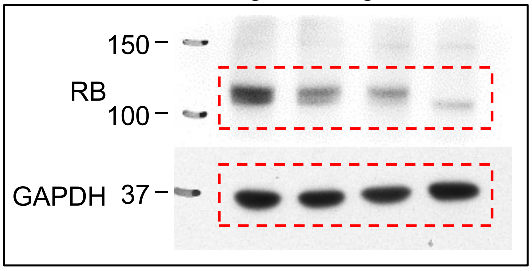

Full unedited gel for Figure 1I

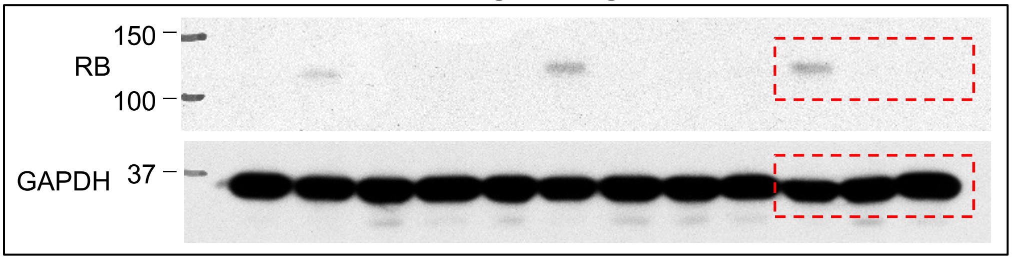

Full unedited gel for Figure 1L

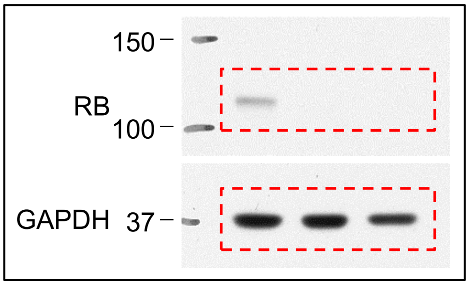

Full unedited gel for Figure 1O

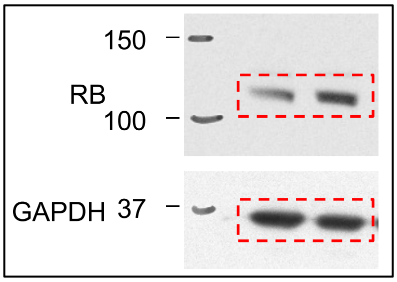

Full unedited gel for Figure 2D

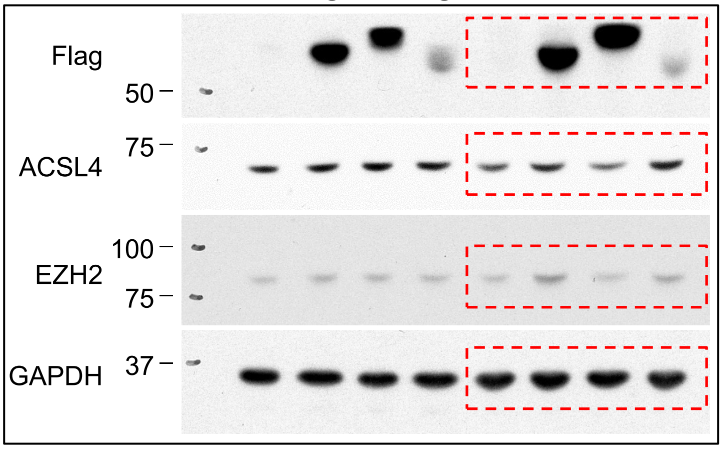

Full unedited gel for Figure 2I

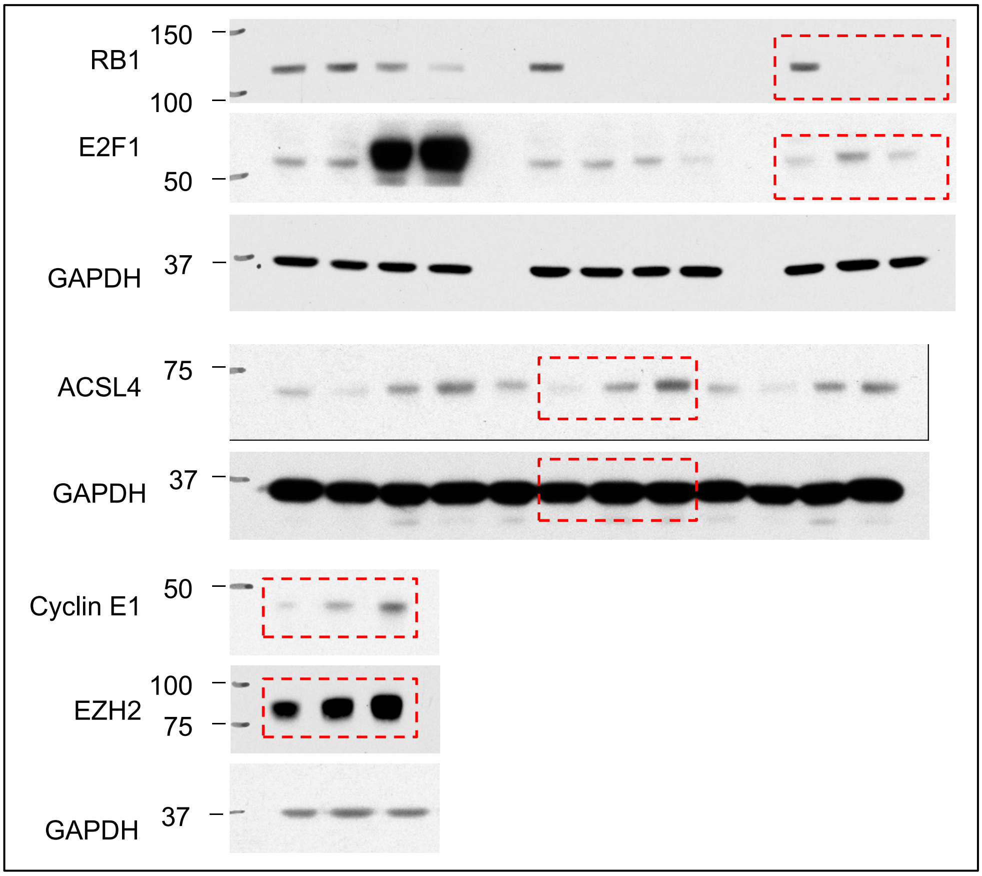

Full unedited gel for Figure 2J

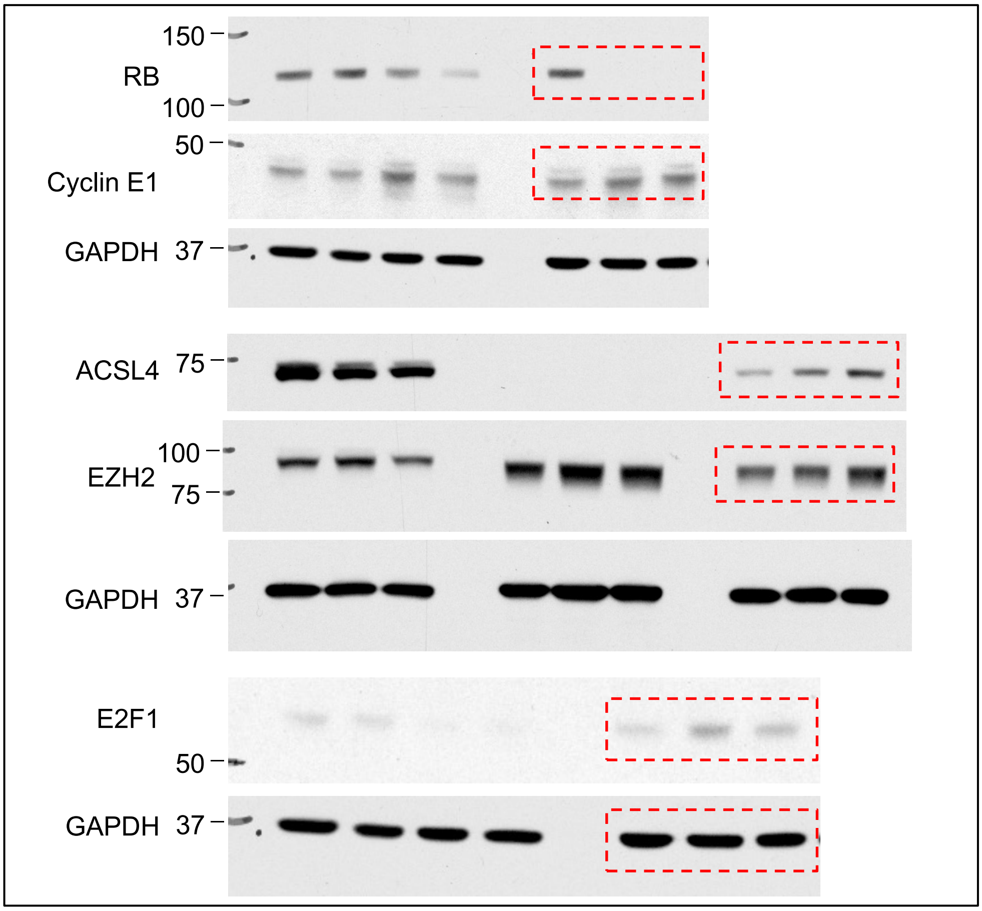

Full unedited gel for Figure 2K

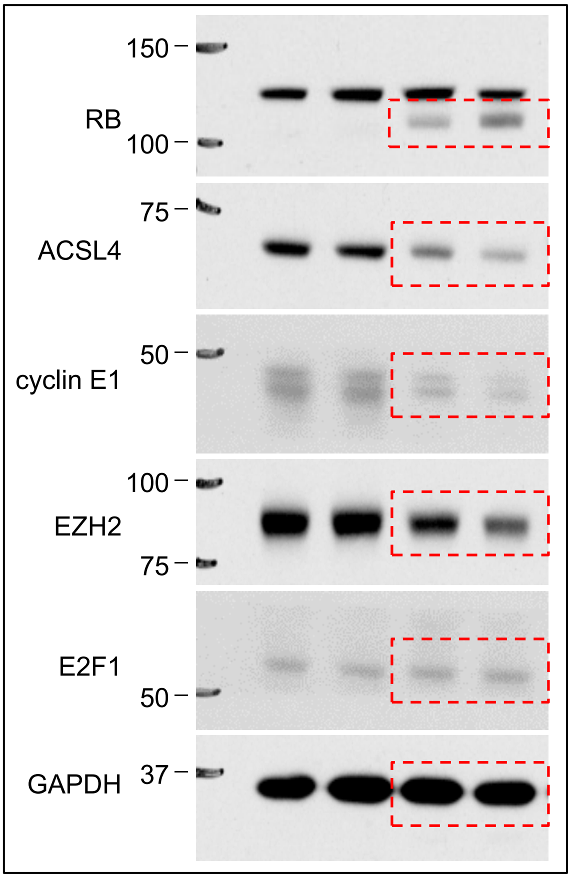

Full unedited gel for Figure 2L

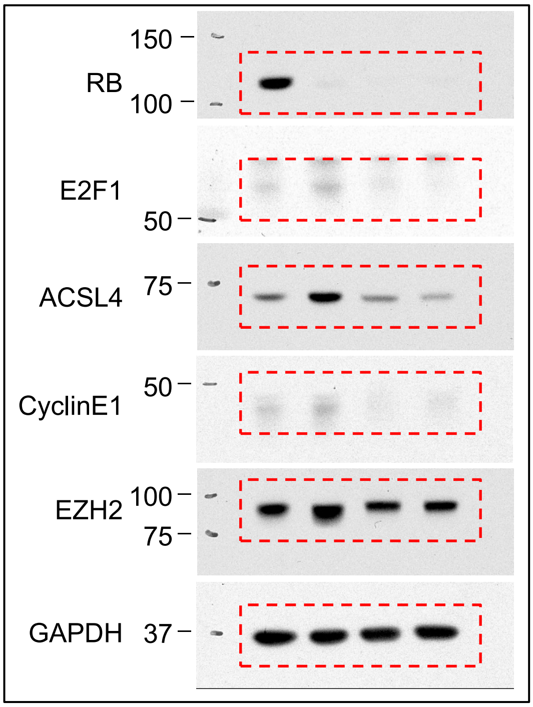

Full unedited gel for Figure 3B

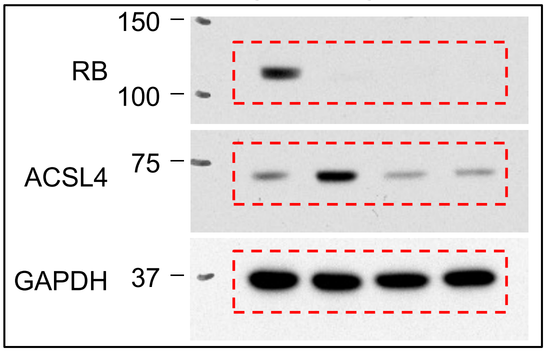

Full unedited gel for Supplemental Figure 1B

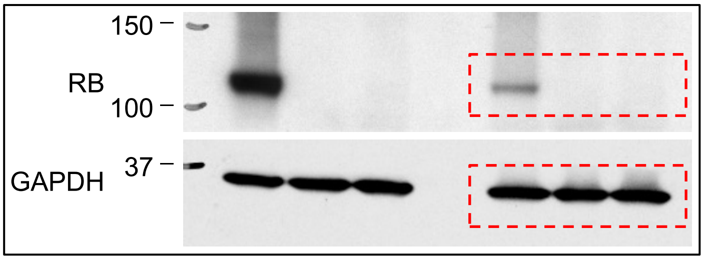

Full unedited gel for Supplemental Figure 1D

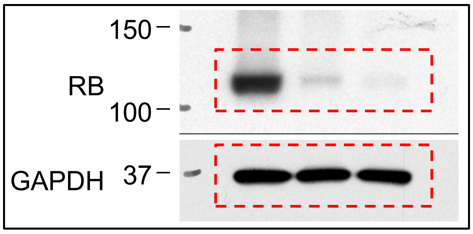

Full unedited gel for Supplemental Figure 1F

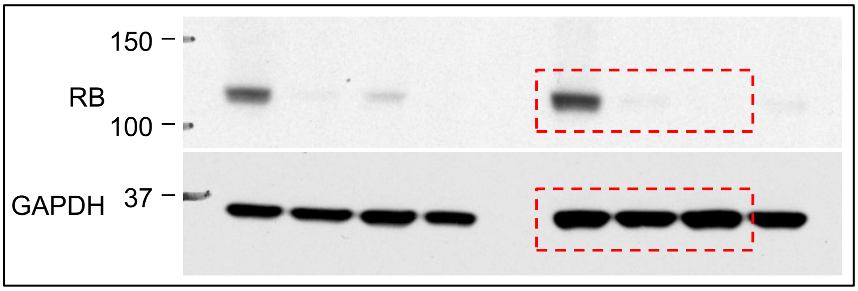

Full unedited gel for Supplemental Figure 2A

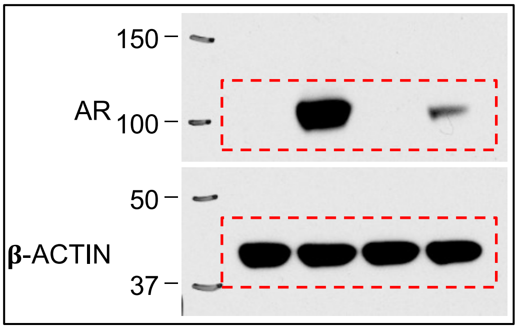

Full unedited gel for Supplemental Figure 2D

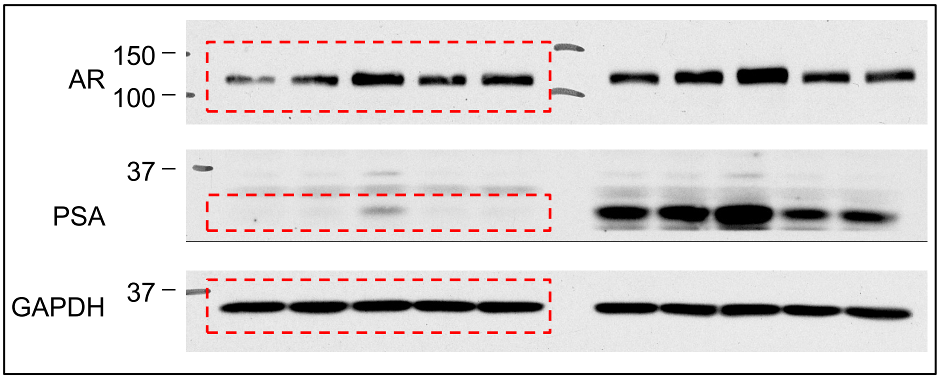

Full unedited gel for Supplemental Figure 3H

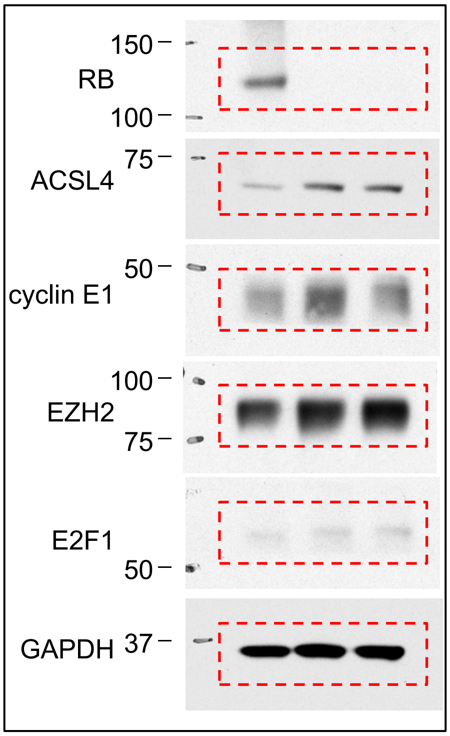

Full unedited gel for Supplemental Figure 2E

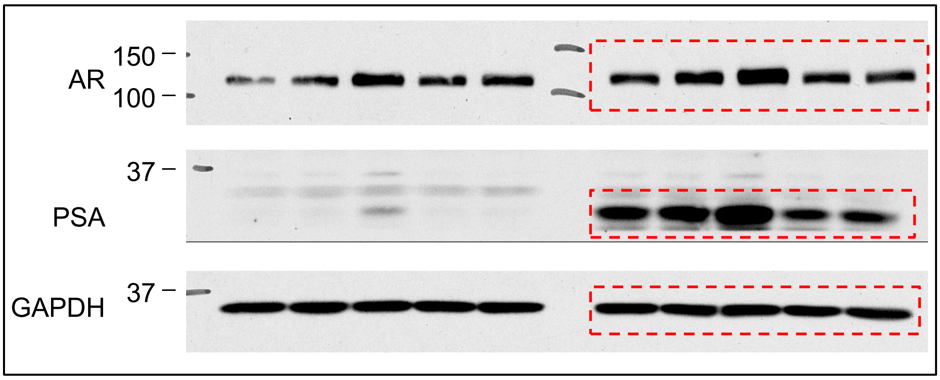

Full unedited gel for Supplemental Figure 3G

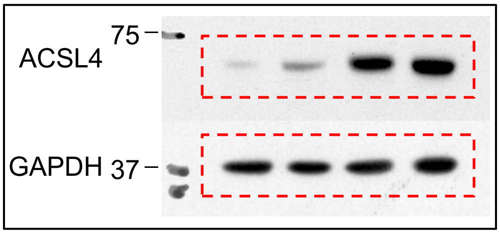

Full unedited gel for Supplemental Figure 3I

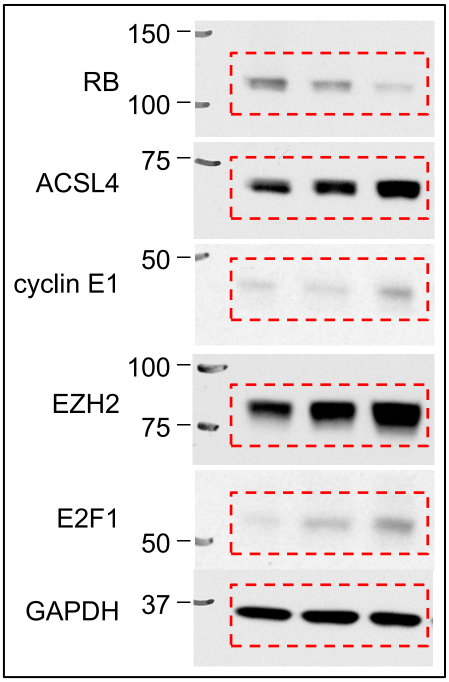

Full unedited gel for Supplemental Figure 3J

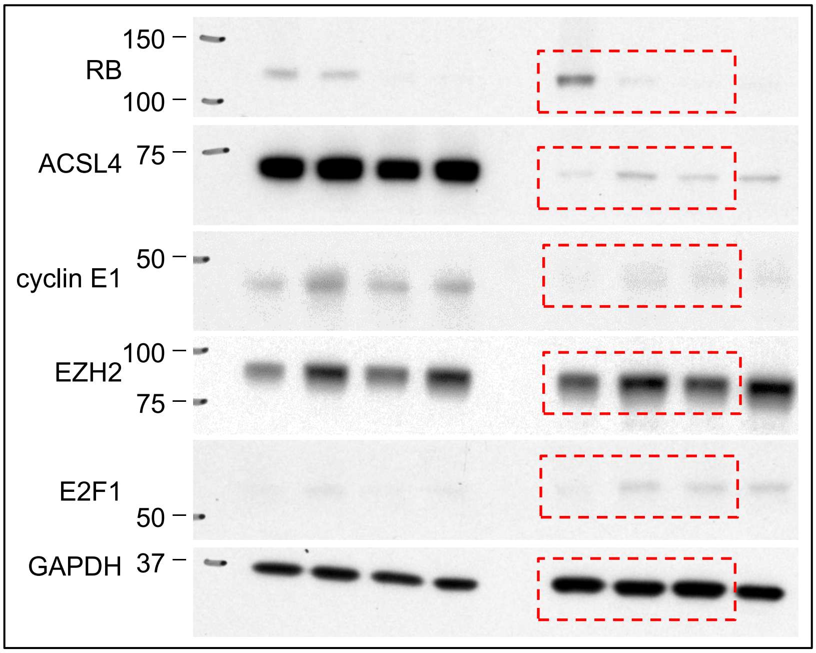

Full unedited gel for Supplemental Figure 4E

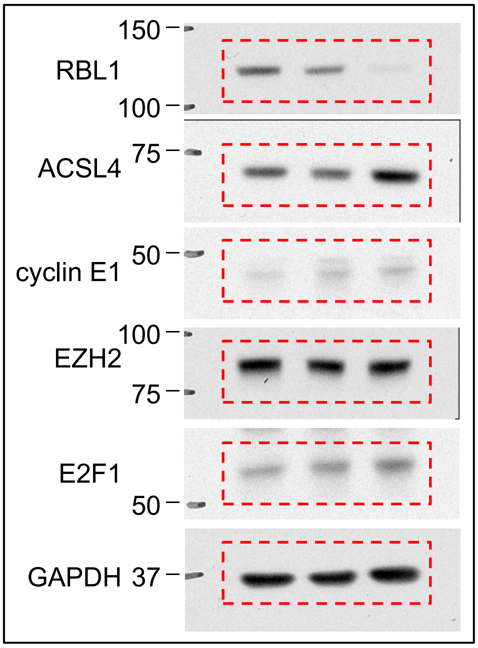

Full unedited gel for Supplemental Figure 4G

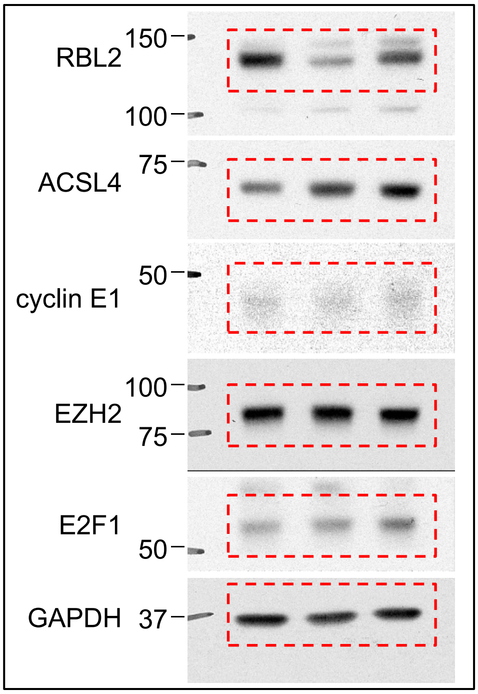

Supplement: Supplemental data [file jci-133-166647-s023.pdf]
